# Management of Low and Intermediate Risk Adult Rhabdomyosarcoma: A Pooled Survival Analysis of 553 Patients

**DOI:** 10.1038/s41598-018-27556-1

**Published:** 2018-06-19

**Authors:** Maha A. T. Elsebaie, Mohamed Amgad, Ahmed Elkashash, Ahmed Saber Elgebaly, Gehad Gamal E. l. Ashal, Emad Shash, Zeinab Elsayed

**Affiliations:** 1 Faculty of Medicine, Ain Shams University, Cairo, Egypt; 2 Department of Biomedical Informatics, Emory University School of Medicine, Atlanta, GA USA; 3 Kasr Al Ainy School of Medicine, Cairo University, Cairo, Egypt; 4 Faculty of Medicine, Al-Azhar University, Cairo, Egypt; 5 Medical Research Education and Practice Association (MREP), Cairo, Egypt; 6 Medical Oncology Department, National Cancer Institute, Cairo University, Cairo, Egypt; 7 Adult Sarcoma Division, Clinical Oncology Department, Ain Shams University Hospitals, Cairo, Egypt

## Abstract

This is the second-largest retrospective analysis addressing the controversy of whether adult rhabdomyosarcoma (RMS) should be treated with chemotherapy regimens adopted from pediatric RMS protocols or adult soft-tissue sarcoma protocols. A comprehensive database search identified 553 adults with primary non-metastatic RMS. Increasing age, intermediate-risk disease, no chemotherapy use, anthacycline-based and poor chemotherapy response were significant predictors of poor overall and progression-free survival. In contrast, combined cyclophosphamide-based, cyclophosphamide + anthracycline-based, or cyclophosphamide + ifosfamide + anthracycline-based regimens significantly improved outcomes. Intermediate-risk disease was a significant predictor of poor chemotherapy response. Overall survival of clinical group-III patients was significantly improved if they underwent delayed complete resection. Non-parameningeal clinical group-I patients had the best local control, which was not affected by additional adjuvant radiotherapy. This study highlights the superiority of chemotherapy regimens –adapted from pediatric protocols- compared to anthracycline-based regimens. There is lack of data to support the routine use of adjuvant radiotherapy for non-parameningeal group-I patients. Nonetheless, intensive local therapy should be always considered for those at high risk for local recurrence, including intermediate-risk disease, advanced IRS stage, large tumors or narrow surgical margins. Although practically difficult (due to tumor’s rarity), there is a pressing need for high quality randomized controlled trials to provide further guidance.

## Introduction

Rhabdomyosarcoma (RMS) is a highly-malignant soft tissue sarcoma. It is a typical tumor of childhood and a rare tumor in adults^1,2^. The rarity of adult RMS hindered accrual to randomized controlled trials, which in turn resulted in a lack of established treatment guidelines. Consequently, there exists an ongoing controversy on how to best manage these patients, and whether a modification of protocols for pediatric RMS, adult soft-tissue sarcoma or custom regimens should be adopted. While substantial improvements were achieved in the survival of pediatric patients with 5-year overall survival (5y-OS) rates between 77–87%^3^, the 5y-OS rates of adult patients remains significantly worse at 20–40%^2,4^. Many potential explanations for the dismal survival were proposed through retrospective analyses, including age, higher incidence of unfavorable tumor sites, higher rates of alveolar/pleomorphic/undifferentiated histologies, and higher IRS-stage^1,5^. Other analyses raised concerns that adults sometimes did not receive chemotherapy, or received lesser dose-intensities^2,6,7^. Recent studies have also shown that adults treated with multidisciplinary approaches -adopted from pediatric protocols-, often have significantly better outcomes, although not as good as pediatric patients^2,6,8,9^.

Through systematic analysis of published cases, we curated and analyzed a dataset consisting of 553 non-metastatic adult RMS patients. To the best of our knowledge, this is the second-largest pooled survival analysis of adult RMS patients so far; the first was a 2009 SEER database analysis of 617 patients^1^. Some data essential for management analysis, including use of chemotherapy, were not recorded in the SEER database, which prevented the investigators from addressing pertinent therapeutic details. The primary objectives of this study include: (1) Identifying prognosticators that can explain the poor survival of adult RMS; (2) Investigating the role of different chemotherapeutic regimens in improving overall and recurrence-free survival; (3) Exploring the local-control benefit of delayed surgery and adjuvant radiotherapy.

## Methodology

### Inclusion and exclusion criteria

Studies considered had to include adults >16 years old (y.o) with primary, histopathologically-proven, non-metastatic RMS. Information had to be disclosed for individual patients rather than for groups. Excluded studies included: (1) Clinicopathological, ultra-structural, and genetic studies; (2) Studies describing rhabdomyosarcoma as a teratomatous tumor or mixed tumor (e.g. carcinosarcoma).

### Database search strategy

**Figure 1:**
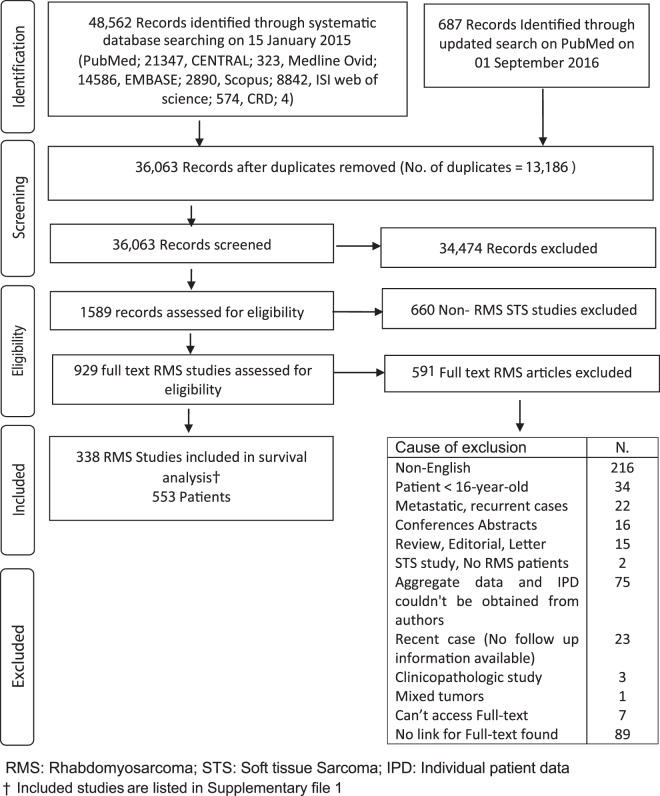
PRISMA flowchart.

We conducted a comprehensive search on PubMed, Medline-Ovid, Cochrane library, EMBASE, Scopus and ISI web of science (Fig. 1)^10^. *Due to space constraints, all included studies along with the detailed search strategies were outlined in* Supplementary S1. Reference lists of all included articles were hand-searched. All prospective/retrospective, published/unpublished articles written in English language were considered.

### Data extraction

A comprehensive data extraction form (Supplementary S2) was used to collect information on patient demographics, clinicopathological characteristics, allocated interventions and follow-up. A comprehensive appendix containing definitions, guidelines and color codes used for data extraction can be found in Supplementary S3. For example, Clinical group and Risk group were defined according to the Intergroup Rhabdomyosarcoma Study Group (IRSG) risk stratification guidelines^11^. Four reviewers did data extraction, all cases were double-checked and any discrepancy resolved by consensus. Because of the long time-span of the included studies, a wide variety of chemotherapy regimens were used. To allow for meaningful comparisons, we devised a categorization system (Supplementary Fig. S4.1) to classify chemotherapy into distinct groups. Quality assessment was based on the clarity, availability and individualization of reported data in original articles (Supplementary Table S4.2). If reports were deficient in one or more subjects (intermediate quality), the corresponding author was contacted twice asking for the missing data.

### Definition of endpoints

The events considered were death (OS), local recurrence (Local Recurrence-Free Survival, LRFS), distant metastasis (Distant Metastasis-Free Survival-DMFS) or both (Progression-Free Survival-PFS). Refractory cases whose primary tumors didn’t demonstrate response to treatment (i.e. who had no tumor-free period at the primary site) were excluded from the local recurrence analysis, but were included in the PFS analysis if their disease progressed regionally or distally.

### Statistical analysis

#### Survival probabilities

Cumulative probabilities were estimated using the Kaplan-Meier method^12^. The log-rank test was used to compare survival of patient subgroups. All analyses were performed using MATLAB (v.R2016b, The Mathworks Inc., USA), R software (*survival* and *survminer* packages) and IBM SPSS-22.0. The cox proportional-hazards (PH) regression models were performed using MATLAB’s in-built *‘coxphfit’* function. Differences in cumulative survival probabilities were compared using the Wilcoxon statistic. Differences in the distribution of categorical variables were compared using the Chi-square or two-sided Fisher exact tests. Non-parametric Mann-Whitney or Kruskal-Wallis tests were used to compare differences between continuous variables. P-values were considered significant at *p* < *0.05*.

#### Patient set assignment

25% of the cohort was randomly withheld as a testing set that was not involved in feature selection or the survival model training and was only used for assessing model generalizability. After feature selection and assessment of testing model accuracy, the entire cohort was used to obtain the final set of models and hazard ratios (HR) presented in the current work.

#### Cox PH regression models

To be considered for the multivariate analysis, variables had to meet the hazard-proportionality assumption and to be significant at the univariate level, using a significance level of p < 0.01 for OS/PFS and p < 0.05 for LRFS/DMFS. The difference in significance level (used for feature selection) is attributed to missing outcomes, which were more frequent with LRFS/DMFS. To ensure robustness of the multivariate models, a minimum threshold of 13 events per model covariate was set. Hence, four covariates were selected for each OS/PFS multivariate model and three covariates for LRFS/DMFS. Predictors were selected based on the absolute value of the univariate model coefficients, given that they meet the significance level. Patient age was included in all multivariate models whenever it met the significance threshold.

#### Model accuracy assessment

Model accuracy and generalizability were measured using Harrell’s Concordance Index (C-index), which is a non-parametric measure of the proportion of orderable patient pairs whose order was correctly predicted by the survival model. C-index ranges between 0 and 100%, where 50% represents random chance and 100% represents perfect classification^13^.

### Data availability statement

Full patient dataset is available in Supplementary S2.

### Ethical disclosure

This article does not contain any experiments with human participants or animals performed by any of the authors. All research data were obtained from already published case reports and case series on bibliographic databases, hence no ethical approval or informed consents were required for conducting the research.

## Results

### Patient and tumor characteristics

A total of 553 patients were included. Patient and tumor characteristics are summarized in Table 1. Patients’ ages ranged from 16–87 y.o (median, 30 y.o). Tumor size was available for 291 patients (52.6%) and ranged from 1–54 cm (median, 6 cm) (Table 1).

**Table 1:** Patient and tumor related characteristics affecting overall and local recurrence-free survival outcomes.

Characteristics	N.	%	Overall Survival (OS)						Local Recurrence Free survival (LRFS)					
			Univariate	Multivariate analysis					Univariate	Multivariate analysis				
			p-value	Patients	Events	HR	95% CI	Test C-index	p-value	Patients	Events	HR	95% CI	Test C-index
Age (yr)	553	100	**<0.001** ^**a**^	253	73	1.25	0.99–1.57	76%	**<0.001**	320	73	**1.36**	**1.08–1.708**	**72%**
Tumor size (cm)	291	52.6	**<0.001**	155	43	1.18	0.98–1.41	81%	**0.029**	172	43	1.15	0.93–1.407	79%
Gender (Male)	281	50.8	0.171	241	67	1.47	0.89–2.4	76%	0.151	314	73	0.73	0.45–1.16	71%
**Tumor site**														
Head/Neck (Non PM)	95	17.2	**0.011**	253	73	0.78	0.35–1.68	76%	0.084	320	73	**0.33**	**0.14–0.782**	**73%**
Extremities	43	7.8	0.187	253	73	1.79	0.87–3.65	77%						
GU (Non B/P)	195	35.3	**<0.001**	253	73	0.85	0.42–1.72	76%	**0.016**	320	73	1.10	0.605–2.00	69%
GU (B/P)	33	6.0	**<0.001**	253	73	3.00	0.89–10.0	76%	0.198	320	73	2.10	0.81–5.4	72%
Head/Neck (PM)	97	17.5							0.267	320	73	1.32	0.69–2.5	70%
Orbit	17	3.1							0.904	320	73	1.03	0.25–4.24	72%
Others ^**b**^	72	13.0												
**Histopathological subtype**														
Botryoides/Spindle cell	88	15.9	0.032	253	73	1.74	0.71–4.23	76%	0.559	320	73	2.18	1.02–4.636	72%
Embryonal	200	36.2	**0.001**	253	73	0.85	0.48–1.49	76%	**0.014**	320	73	0.95	0.52–1.705	70%
Alveolar	99	17.9	**0.001**	253	73	0.87	0.49–1.53	76%	0.086	320	73	1.21	0.62–2.34	71%
Pleomorphic	86	15.6	**0.011**	253	73	0.91	0.45–1.82	75%	0.177	320	73	0.58	0.29–1.11	73%
Undifferentiated/NOS	30	5.4												
**IRS stage**														
Stage I	305	55.2	**<0.001**	239	69	0.67	0.34–1.31	80%	**0.001**	287	61	**0.50**	**0.280–0.89**	**73%**
Stage II	46	8.3	**0.047**	239	69	1.39	0.76–2.51	80%	**0.001**	287	61	**2.54**	**1.31–4.894**	**73%**
Stage III	117	21.2												
**UICC stage**														
UICC stage I	167	30.2	**<0.001**	253	73	0.82	0.47–1.40	76%	0.125	226	54	1.10	0.595–2.02	70%
UICC stage II	99	17.9	**0.021**	253	73	1.05	0.58–1.87	76%	0.253	226	54	0.99	0.535–1.81	70%
UICC stage III	87	15.7												
Tumor status	356	64.4	**<0.001**	235	69	1.19	0.49–2.8	77%	0.063	224	56	1.01	0.57–1.78	70%
Nodal status	439	79.4	**0.002**	252	72	0.94	0.52–1.68	76%	0.628	273	64	0.78	0.39–1.52	74%
**IRS Risk group**														
Intermediate risk	255	46.1	**<0.001**	253	73	**3.28**	**1.76–6.11**	**76%**	**<0.001**	320	73	**2.61**	**1.47–4.64**	**72%**
low risk	216	39.1												

^a^**Bold = **significant values (p < 0.05); ^b^others include thoracic, abdominal and retroperitoneal tumor sites; PM: para-meningeal; GU: Genitourinary; B/P: Bladder/Prostate; IRS: Intergroup Rhabdomyosarcoma Study; UICC: International Union Against Cancer TNM staging guidelines; For the multivariate models, each variable was adjusted to the following set of covariates: **OS analysis:** Age, UICC stage I, GU (B/P), IRS Post-Surgical Group-I and IRS risk group; **LRFS analysis:** Age, IRS Post-Surgical Group-I, IRS Post-Surgical Group-II and IRS risk group. Empty rows correspond to covariates that were not entered into the model because they were either explained by other covariates or did not meet the model inclusion criteria (due to small number of events or other reasons - see Methodology section for details).

### Treatment overview

The most frequent therapeutic modality was surgery (SUR) and chemotherapy (CT) (27.5%). The 5 y survival rates (OS, LRFS, PFS, in order) for the different treatment modalities were as follows: 1) *Trimodal therapy:* 53%, 70%, 57%; 2) *SUR* + *CT:* 69%, 76%, 67%*; 3) SUR* + *XRT*: 59%, 63%, 48%; *4) SUR alone:* 43%, 59%, 38%; *5) XRT* + *CT:* 36%, 66%, 47%. The Wilcoxon p-value for differences in OS, LRFS and PFS were *p* < *0.001, p* = *0.019, and p* = *0.005*, respectively (Table 2).

**Table 2:** Treatment related characteristics affecting overall and local recurrence-free survival outcomes.

Characteristics	N.	%	Overall Survival (OS)						Local Recurrence Free survival (LRFS)					
			Univariate	Multivariate analysis					Univariate	Multivariate analysis				
			p-value	Patients	Events	HR	95% CI	Test C-index	p-value	Patients	Events	HR	95% CI	Test C-index
**IRS Post-Surgical Group**														
Group I	173	31.3	** < 0.001**	253	73	**0.41**	**0.21–0.77**	**76%**	** < 0.001**	320	73	**0.47**	**0.261–0.84**	**72%**
Group I-II	57	10.3							0.129	320	73	0.63	0.307–1.30	72%
Group II	55	9.9							**0.018**	320	73	1.79	0.975–3.26	72%
Group III	168	30.4												
**Treatment Modality**														
Trimodality	139	25.1	0.225	253	73	0.82	0.47–1.40	76%	0.437	320	73	0.74	0.415–1.30	70%
Surgery and chemotherapy	152	27.5	**0.001**	253	73	0.99	0.54–1.79	76%	0.070	320	73	0.97	0.551–1.72	71%
Surgery alone	105	19.0	0.067	253	73	0.94	0.44–2.01	77%	0.096	320	73	0.98	0.520–1.84	71%
Surgery and radiotherapy	62	11.2	0.953	253	73	1.24	0.55–2.77	76%	0.588	320	73	0.98	0.465–2.05	71%
Radio and Chemotherapy	73	13.2							0.869	320	73	1.17	0.57–2.382	71%
Chemo Only or Radio Only	21	3.8												
Chemotherapy Use	377	68.2	**0.023**	253	73	0.89	0.46–1.70	76%	**0.013**	320	73	0.79	0.45–1.367	70%
**Category of Chemotherapy**														
Cyclo based	84	22.5	0.593	185	55	1.51	0.71–3.18	73%	0.746	224	46	1.39	0.68–2.855	70%
Cyclo and Anthracycline based	79	21.2	**0.002**	185	55	0.54	0.24–1.21	72%	**0.017**	224	46	0.46	0.191–1.11	73%
Ifo and Anthracycline based	52	13.9	0.443	185	55	1.46	0.75–2.82	75%	0.999	224	46	1.23	0.536–2.80	71%
Anthracycline based	18	4.8	**0.012**	185	55	1.68	0.50–5.53	74%	0.076	224	46	**2.77**	**1.059–7.24**	**70%**
Ifo based	15	4.0												
Cyclo and Ifo and Anthracycline	15	4.0												
Vincristine/VA only	33	8.8												
Unknown	77	20.6												
**Timing of Chemotherapy**														
Neoadjuvant	26	5.1	**0.040**	247	70	0.55	0.15–1.90	76%						
Adjuvant	219	42.9	** < 0.001**	247	70	1.10	0.66–1.84	75%	**0.039**	312	70	0.83	0.490–1.41	71%
Both pre and post-surgery	15	2.9	0.059	247	70	0.23	0.03–1.63	76%						
Primary modality	78	15.3							0.634	312	70	1.16	0.572–2.36	72%
**Response to chemotherapy**														
Complete response (CR)	51	9.2	** < 0.001**	250	72	**0.18**	**0.05–0.58**	**79%**	**0.007**	317	73	**0.24**	**0.073–0.78**	**73%**
Disease progression (PD)	35	6.3	** < 0.001**	250	72	**4.34**	**2.23–8.41**	**80%**	** < 0.001**	317	73	3.60	1.567–8.28	73%
Partial response (PR)	34	6.1							**0.013**	317	73	**2.21**	**1.096–4.47**	**70%**
Stable disease (SD)	8	1.4												
Not available (N/A)	87	15.7												
Not applicable	334	60.4												

^a^**Bold** = significant values (p < 0.05); Cyclo: Cyclophosphamide; Ifo: Ifosfamide; VA: Vincristine, Actinomycin-D. For the multivariate models, each variable was adjusted to the following set of covariates: **OS analysis:** Age, UICC stage I, GU (B/P), IRS Post-Surgical Group-I and IRS risk group; **LRFS analysis:** Age, IRS Post-Surgical Group-I, IRS Post-Surgical Group-II and IRS risk group. Empty rows correspond to covariates that were not entered into the model because they were either explained by other covariates or did not meet the model inclusion criteria (due to small number of events or other reasons - see Methodology section for details).

Local-control consisted of SUR in 256 patients (46.3%), SUR and XRT in 200 patients (36.2%) and XRT alone in 81 patients (14.6%). Radiation doses were available for 159 (57%) of the 281 patients who received XRT (with or without SUR) for local control. The median radiation doses for tumors locally controlled with SUR and XRT vs. those locally controlled with XRT alone were 54 Gy (range 14–110, IQR = 14.0) vs. 56.5 Gy (range 36–110, IQR = 11.8), *p* = 0.319.

377 patients (68.2%) received CT. The reasons why not all patients received CT couldn’t be retrieved in all cases; some patients were lost to follow up, while others refused CT or were in a poor clinical condition impairing CT administration. 37.9% (36 out of 95) of the elderly patients (≥60 y.o) received chemotherapy, compared to 74.4% (337 out of 453) of the young and middle aged adults (<60 y.o), *p* < 0.001. Young and middle-aged adult patients constituted 90.3% (n = 337) of all patients who received chemotherapy in our dataset (n = 377).

### Pretreatment features affecting survival outcomes

**Figure 2:**
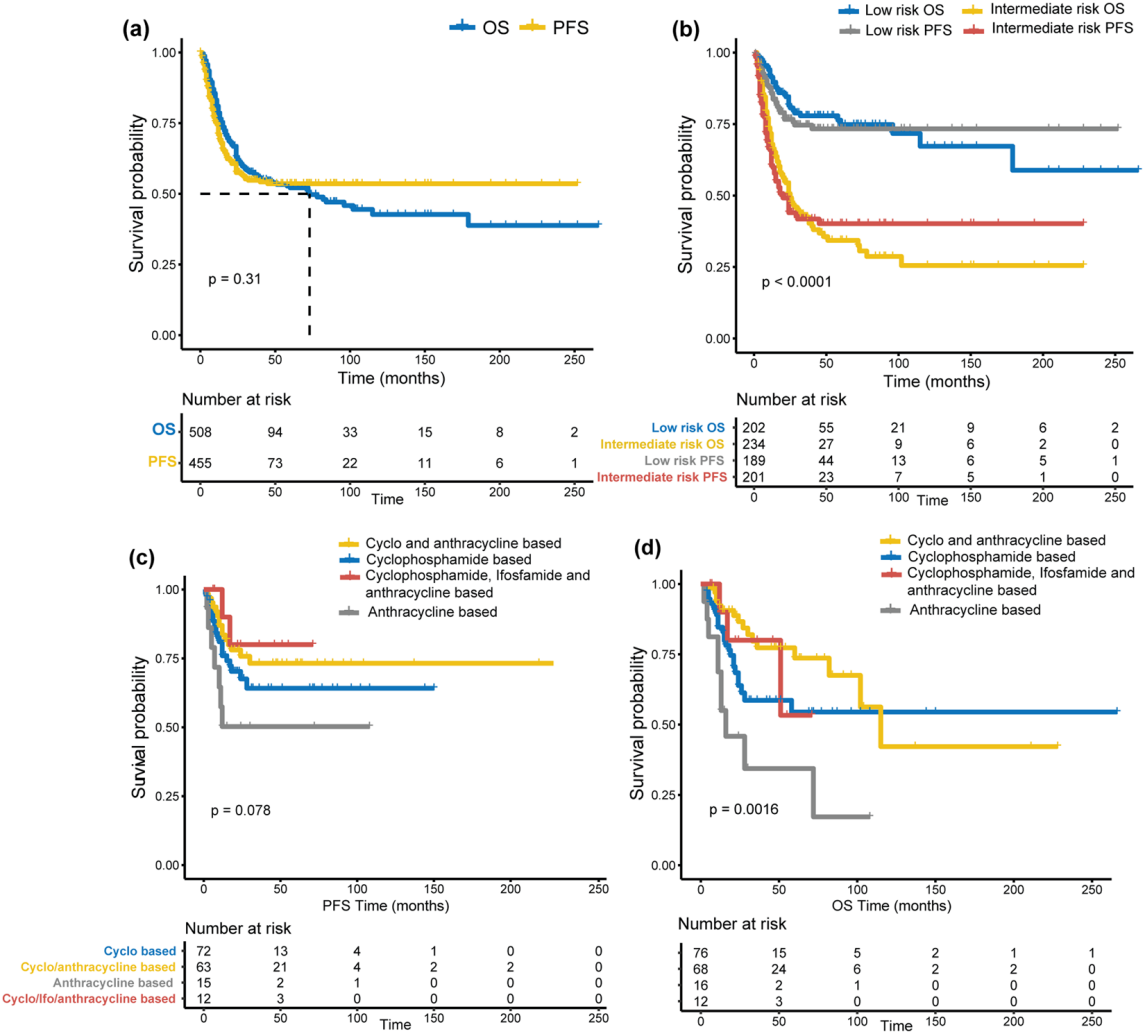
(**a**) Kaplan-Meier (KM) OS and PFS curves for the whole cohort; (**b**) KM OS and PFS curves for the whole cohort according to the IRS risk group (low risk vs. intermediate risk); (**c**) Comparative efficacy of different chemotherapeutic regimens on PFS outcomes; (**d**) Comparative efficacy of different chemotherapeutic regimens on OS outcomes.

The 5 y and 10y-OS rates were 52.7% and 42.7%, respectively, with a median survival time of 74 months (range, 1–266 months). The 5 y and 10y-PFS rates were 53.5% (Fig. 2a). Risk of disease progression increased by 1.32 for every unit (year) increase in age. (Table 3) Low-risk patients had better 5y-OS and PFS rates (77% and 73%) than intermediate-risk patients (35% and 40% respectively, *p* < 0.001) (Fig. 2b). There was a notable difference in the distribution of different age groups between low and intermediate-risk patients. (Supplementary Fig. S4.3) However, this age difference did not affect prognosis of intermediate-risk patients. Head/neck (non-PM) tumors (n = 95) were associated with a 68% reduction in the risk of tumor progression compared to other tumor sites. (Table 3)

**Table 3:** Patient and tumor related characteristics affecting distant metastasis and progression-free survival outcomes.

Characteristics	Distant Metastasis Free Survival (DMFS)						Progression Free survival (PFS)					
	Univariate	Multivariate analysis					Univariate	Multivariate analysis				
	p-value	Patients	Events	HR	95% CI	Test C-index	p-value	Patients	Events	HR	95% CI	Test C-index
Age (yr)	** < 0.001** ^**a**^	390	94	1.13	0.92–1.373	61%	** < 0.001**	342	113	**1.32**	**1.082–1.61**	**70%**
Tumor size (cm)	**0.008**	204	52	1.06	0.845–1.327	62%	**0.008**	178	58	1.03	0.85–1.25	69%
Gender (Male)	0.091	380	93	1.50	0.98–2.286	67%	0.987	333	112	1.14	0.782–1.66	71%
**Tumor Site**												
Head/Neck (Non PM)	** < 0.001**	390	94	**0.18**	**0.055–0.565**	**61%**	**0.001**	342	113	**0.32**	**0.153–0.681**	**70%**
Orbit	0.817	390	94	0.96	0.30–3.044	61%	0.452	342	113	0.70	0.22–2.218	70%
GU (Non B/P)	0.126	390	94	1.16	0.70–1.894	61%	**0.005**	342	113	1.08	0.645–1.798	69%
GU (B/P)	**0.002**	390	94	2.11	0.89–4.94	61%	** < 0.001**	342	113	1.91	0.889–4.11	70%
Extremities	0.079	390	94	0.82	0.39–1.727	62%	0.207	342	113	1.22	0.55–2.65	70%
Head/Neck (PM)							0.055	342	113	0.87	0.53–1.428	69%
**Histopathological subtype**												
Botryoides/Spindle cell	**0.046**	389	93	1.14	0.54–2.39	60%	0.234	341	112	1.65	0.90–2.99	68%
Embryonal	**0.003**	389	93	1.02	0.628–1.66	60%	**0.001**	341	112	0.78	0.499–1.2117	69%
Alveolar	**0.044**	389	93	0.91	0.53–1.539	59%	**0.032**	341	112	1.03	0.621–1.713	69%
Pleomorphic	0.080	389	93	0.80	0.46–1.383	60%	0.161	341	112	0.79	0.429–1.44	70%
**IRS stage**												
Stage I	** < 0.001**	347	75	1.17	0.694–1.98	62%	** < 0.001**	302	91	0.92	0.55–1.538	68%
Stage II	0.741	347	75	0.64	0.29–1.413	63%	0.058	302	91	1.39	0.760–2.558	68%
**UICC stage**												
UICC stage I	** < 0.001**	265	59	**0.39**	**0.206–0.722**	**69%**	** < 0.001**	240	77	0.87	0.466–1.604	70%
UICC stage II	0.027	265	59	1.22	0.701–2.137	60%	**0.013**	240	77	1.03	0.624–1.685	70%
Tumor status	** < 0.001**	268	66	**1.93**	**1.127–3.313**	**67%**	** < 0.001**	239	80	1.38	0.80–2.35	70%
Nodal status	**0.001**	324	69	**2.01**	**1.184–3.398**	**62%**	0.070	287	90	1.01	0.60–1.689	70%
IRS Risk group	** < 0.001**	390	94	**3.37**	**2.05–5.536**	**61%**	** < 0.001**	342	113	**1.96**	**1.237–3.115**	**70%**

^a^**Bold = **significant values (p < 0.05); PM: para-meningeal; GU: Genitourinary; B/P: Bladder/Prostate; IRS: Intergroup Rhabdomyosarcoma Study; UICC: International Union Against Cancer TNM staging guidelines; For the multivariate models, each variable was adjusted to the following set of covariates: **DMFS analysis:** Age, Head/Neck (Non PM) tumor site, GU (B/P) tumor site, and IRS risk group; **PFS analysis:** Age, Head/Neck (Non PM) tumor site, IRS Post-Surgical Group-I, IRS Post-Surgical Group-II and IRS risk group. Empty rows correspond to covariates that were not entered into the model because they were either explained by other covariates or did not meet the model inclusion criteria (due to small number of events or other reasons - see Methodology section for details).

### Patients at risk for distant-metastasis and disease progression

Tumor invasiveness and lymph-node involvement at diagnosis were associated with a two-fold increase in the risk of distant-metastasis (Table 3). Risk of disease progression decreased by 48% if patients received multi-agent chemotherapy. (Table 4) (Fig. 2a).

**Table 4:** Treatment related characteristics affecting distant metastasis and progression-free survival outcomes.

Characteristics	Distant Metastasis Free Survival (DMFS)						Progression Free survival (PFS)					
	Univariate	Multivariate analysis					Univariate	Multivariate analysis				
	p-value	Patients	Events	HR	95% CI	Test C-index	p-value	Patients	Events	HR	95% CI	Test C-index
**IRS Post-surgical Group**												
Group I	** < 0.001**	342	78	**0.40**	**0.225–0.703**	**70%**	** < 0.001**	342	113	**0.34**	**0.212–0.554**	**70%**
Group I-II	0.038	342	78	1.57	0.827–2.974	66%	0.033	342	113	0.86	0.491–1.519	70%
Group II	0.068	342	78	**2.16**	**1.21–3.83**	**65%**	**0.020**	342	113	1.25	0.736–2.13	70%
**Treatment Modality**												
Trimodality	0.246	390	94	0.69	0.419–1.14	64%	0.321	342	113	0.82	0.52–1.284	68%
Surgery and Chemotherapy	**0.015**	390	94	0.65	0.386–1.106	64%	**0.004**	342	113	0.72	0.44–1.191	69%
Radio and Chemotherapy	0.251	390	94	1.19	0.673–2.119	62%	0.509	342	113	0.98	0.585–1.652	69%
Surgery alone	0.028	390	94	1.09	0.633–1.89	62%	**0.012**	342	113	1.04	0.587–1.825	70%
Surgery and Radiotherapy	0.907	390	94	**2.19**	**1.192–4.01**	**64%**	0.498	342	113	**2.01**	**1.0783–3.761**	**69%**
Chemotherapy Use	**0.026**	390	94	**0.61**	**0.382–0.9708**	**66%**	**0.001**	342	113	**0.52**	**0.315–0.859**	**70%**
**Category of Chemotherapy**												
Cyclo based	0.572	269	59	1.40	0.72–2.69	64%	0.427	241	74	1.27	0.721–2.218	71%
Cyclo and Anthracycline based	0.231	269	59	0.87	0.431–1.75	63%	**0.021**	241	74	0.56	0.291–1.078	69%
Ifo and Anthracycline based	0.843	269	59	0.92	0.434–1.94	63%	0.704	241	74	0.89	0.446–1.77	70%
Anthracycline only based	0.243	269	59	1.13	0.414–3.087	63%	0.139	241	74	**2.73**	**1.154–6.475**	**70%**
Cyclo and Ifo and Anthracycline based							0.200	241	74	0.40	0.098–1.659	70%
Ifo based												
**Timing of Chemotherapy**												
Adjuvant	**0.006**	371	91	**0.62**	**0.394–0.98**	**64%**	**0.001**	334	110	0.79	0.515–1.208	68%
Both pre and Post surgery	0.223	371	91	0.31	0.043–2.27	58%	0.265	334	110	0.61	0.191–1.963	70%
Primary modality	0.056	371	91	1.36	0.796–2.33	60%	0.366	334	110	0.91	0.541–1.517	69%
Neoadjuvant							0.958	334	110	0.74	0.294–1.84	70%
**Response to chemotherapy**												
Complete response (CR)	**0.017**	387	94	**0.29**	**0.104–0.7906**	**63%**	**0.002**	339	113	**0.25**	**0.11–0.589**	**69%**
Disease progression (PD)	** < 0.001**	387	94	4.36	2.46–7.706	67%	** < 0.001**	339	113	3.50	2.002–6.127	71%
Not available (N/A)	0.628	387	94	1.10	0.580–2.07	61%	0.539	339	113	0.67	0.34–1.295	69%
Partial response (PR)							0.619	339	113	0.91	0.498–1.665	69%
No response (NR)												

^a^**Bold = **significant values (p < 0.05); Cyclo: Cyclophosphamide; Ifo: Ifosfamide; VA: Vincristine, Actinomycin-D. For the multivariate models, each variable was adjusted to the following set of covariates: **DMFS analysis:** Age, Head/Neck (Non PM) tumor site, GU (B/P) tumor site, and IRS risk group; **PFS analysis:** Age, Head/Neck (Non PM) tumor site, IRS Post-Surgical Group-I, IRS Post-Surgical Group-II and IRS risk group. Empty rows correspond to covariates that were not entered into the model because they were either explained by other covariates or did not meet the model inclusion criteria (due to small number of events or other reasons - see Methodology section for details).

The survival benefit of chemotherapy was more evident in PFS compared to OS results. Survival rates for patients who received compared to those who did not receive chemotherapy were: 54% vs. 48%, (p = 0.002), for 5y-OS and 58% vs. 40%, (p = 0.001) for 5y-PFS. On multivariate analysis, the chemotherapy effect on OS was lost, but remained significant in PFS analysis.

Use of anthracycline-only based chemotherapy (no cyclophosphamide/ifosfamide) was associated with a significant high risk of disease progression. (Table 4) In contrast, patients treated with cyclophosphamide-based, cyclophosphamide + anthracycline-based, or cyclophosphamide + ifosfamide + anthracycline-based combinations fared significantly better in terms of 5y-PFS (64%, 74%, 80% vs. 47%; *p* = 0.091, 0.016, 0.037 respectively) and 5y-OS (56%, 78% and 48% vs. 36%; *p* = 0.022, < 0.001, 0.039 respectively). (Fig. 2c and d; Supplementary Table S4.4).

**Figure 3:**
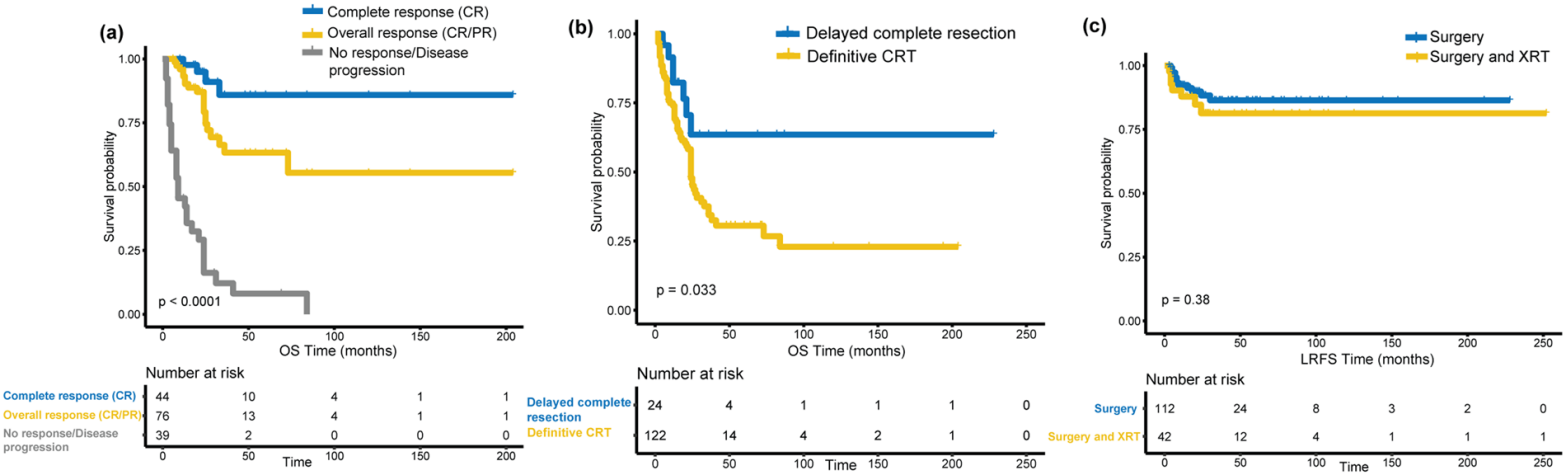
(**a**) KM OS curves for the response to chemotherapy; (**b**) The effect of delayed complete resection on OS of clinical group III patients; (**c**) KM LRFS curves of non-PM group I patients who did vs. who didn’t receive adjuvant radiotherapy.

Assessment of chemotherapy response was possible for 219 patients, 128 of whom had explicitly-reported responses. The overall-response rate (complete/partial response) was 66.4%, with 39.8% achieving complete response (CR). The 5y-OS rate for the CR cohort was 86%. Patients with objective response (complete/ partial response (PR)) had better OS compared to those who achieved no-response (NR) or who experienced disease progression (PD) (5y-OS 66% vs. 8%; p < 0.001). (Fig. 3a) There were no differences in the distributions of type or timing of chemotherapy, histological subtypes or tumor extension between responders and non-responders (*p* = 0.328, 0.95, 0.246, 0.87 respectively). Likewise, tumor size and age were not significantly correlated with chemotherapy response (*p* = 0.788, 0.076 respectively). Conversely, responders to chemotherapy tended to have low-risk tumors (*p* = 0.001) and tumors at favorable sites (*p* = 0.004) (Supplementary Fig. S4.5). The only exception was PM-RMS that showed an overall-response rate of 76.9%. Belonging to the intermediate-risk group retained prognostic significance after including chemotherapy response in the multivariate models of OS (HR 2.69; 95%CI 1.77–4.095; C-index: 72%). Nonetheless, the prognosis of intermediate-risk group patients still improved if their disease responded to chemotherapy (*p* < 0.001) (Supplementary Fig. S4.6).

Among patients who didn’t receive chemotherapy during their course of treatment, there was no significant effect of different tumor histologies or age groups on their overall survival. On the other hand, patients with non-PM, non-orbital head and neck tumor sites fared significantly better compared to other tumor sites (n = 35, 5y-OS = 84%, overall Wilcoxon comparisons = 0.002). Patients with low risk tumors (n = 58) also fared significantly better compared to intermediate risk (n = 78, 5y-OS 66% vs. 34%, p = 0.005).

### Patients at risk for local recurrence

The highest risk of local recurrence was seen in patients with IRS stage-II (unfavorable site), intermediate-risk disease, residual tumor (Group II-III), anthracycline-based regimen and PR to initial CT (Tables 1 and 2). The 5y-OS and LRFS rates for the CR cohort were 85% and 92% compared to 33% and 39% for the PR cohort (*p* < 0.001 for the OS and LRFS comparisons)

Clinical group-III patients were compared based on whether they underwent delayed primary excisions (DPE) or not. The 5y-LRFS for the DPE group (n = 27) compared to the definitive chemoradiotherapy (CRT) group (n = 135) was 68% vs. 60% (*p* = 0.092) while the 5y-OS rates were 66% vs. 32% (*p* = 0.029) (Fig. 3b).

There were no significant differences in the distributions of age groups, tumor site, tumor invasiveness (T status), tumor size or histology between the DPE and the CRT cohorts (p = 0.083, p = 0.788, p = 0.18, p = 0.939, p = 0.056 respectively). 56 (40.6%) patients from the 138 CRT cohort had PM tumor sites compared to 8 (29.6%) patients from the 27 DPE cohort. Likewise, 32 (23.1%) from the CRT cohort had head and neck (non-PM) tumor sites compared to 6 (22.2%) patients from the DPE cohort. The only significant differences between the DPE and CRT groups were in the patients’ response to initial CT/CRT (p < 0.001) and extent of nodal involvement (p = 0.036). 15 (55.6%) patients from the DPE group had radiographic-PR/NR compared to 22 (16%) patients from the CRT group. In contrast, only 2 (4.3%) from group-III patients who achieved radiographic-CR and 1 (3.2%) from group-III patients who achieved radiographic-PD underwent DPE. On a different front, 20 (83.3%) patients from the 27 DPE group had no nodal involvement (N0) compared to 65 (60.7%) patients from the 138 CRT group.

Of the DPE group, 10 patients underwent radical operations (e.g. radical maxillectomy, TAH-BSO, radical cystoprostatectomy), while 16 patients had non-radical approaches (e.g. orbital preserving surgery, wide local excisions). The extent of surgical procedure wasn’t clear in one case. Only six patients had available post-DPE surgical margin status; 4 patients had negative margins (R0) and 2 had microscopically-positive margins (R1). All six patients were alive at a median follow up of 45.5 months (range 12–87).

Both local-control and OS of the group-III PR or NR cohort (n = 42) was significantly improved if patients underwent delayed complete resection (Supplementary Fig. S4.7).

Clinical group-I patients fared significantly better than group II-III with 59% reduction in risk of death and 53% reduction in risk of local progression (Table 2). Non-PM group-I patients (n = 168) had promising 5y-OS of 78% and 5y-LRFS rates of 84%. The 5y-LRFS of non-PM group-I patients who did vs. who did not receive adjuvant XRT was 81% vs. 86% (*p* = 0.448) (Fig. 3c). The only significant difference between controlled and recurrent tumors among non-PM group-I patients was in their risk group distribution. 25% (11/45) of non-PM group-I intermediate-risk patients eventually recurred compared to 10% (11/107) of low risk patients (*p* = 0.04).

## Discussion

The OS results of our cohort are comparable to those reported in other large-scale studies, where the 5y-OS rates for adult non-metastatic patients ranged between 44–55%.(supplementary Table S4.8) Unfavorable clinical presentation with increasing age, as well as age per se are widely-described adverse prognostic factors in adult RMS^1,4–8,14,15^. One analysis comparing the dose-intensities of vincristine/cyclophosphamide/dactinomycin (VAC), found that adults receive significantly lower dose-intensities compared to children, mostly due to high incidence of myelo-suppression, infection, and neurotoxicity^16^. This age variable only exerts influence on patients with loco-regional disease. Conversely, patients with disseminated tumors behave so badly that age does not affect prognosis^6^. This may indicate that adults with localized disease can have better survival outcomes if treated appropriately^2^.

A recent study revealed no significant difference in the 5y-OS rates between non-metastatic children and adolescents treated on four prospective RMS protocols (5y-OS 76.6% vs. 78.6%)^15^.

Due to the tumor’s rarity, the optimal choice of adjuvant therapy remains controversial; VAC is currently the standard regimen for pediatric RMS patients^11^ while anthracycline-based chemotherapy is golden-standard for soft tissue sarcoma patients^17^. We found the use of anthracycline-based chemotherapy (no ifosfamide/cyclophosphamide) was associated with a significant risk of disease progression. In contrast, cyclophosphamide-based, cyclophosphamide + anthracycline-based, or cyclophosphamide + ifosfamide + anthracycline-based regimens yielded significantly better PFS outcomes. In the analysis by Ferrari and colleagues, patients treated with cyclophosphamide/ifosfamide containing regimens (with/without anthracycline) fared better than patients who only received anthracycline-based regimens^2^. Similarly, Little *et al*. reported 10y-OS and DMFS of 47% and 59% for patients treated with VAC or VAC + Anthracycline^18^. Gerber *et al*. reported significantly higher OS-rates for their adult patients treated on pediatric RMS protocols compared to patients treated off-protocol. On-protocol patients were more likely to receive cyclophosphamide, doxorubicin, and vincristine (71% vs. 20%, p < 0.0001)^8^. On a different front, Dumont and colleagues reported poor 5y-PFS of 36% for 163 adolescent/adults with non-metastatic RMS; although most of their patients received chemotherapy, the most commonly-administered regimens were the anthracycline-based (39%)^7^.

In some of the aforementioned adult series, use of chemotherapy (any regimen) for non-metastatic disease was not significant on OS analyses, an observation that was also indicated by our multivariate analysis^7,8^. This could be partially explained by deaths that were not caused by the primary tumor but by other comorbidities.

Furthermore, the role of chemotherapy for adult head and neck rhabdomyosarcoma is controversial. Our analysis identified a small portion of adult patients with localized, non-orbital, non-PM, head and neck RMS who were managed with SUR only or SUR and XRT (n = 35 out of 95 non-orbital non-PM Head and Neck RMS patients, 36%) and had high 5y-OS rates. For head and neck soft tissue sarcomas of adults, surgical resection with wide margins followed by adjuvant radiotherapy is the treatment of choice. Postoperative chemotherapy is then considered for selected patients, at high risk for recurrence^17^. A similar approach has been widely described as the treatment of choice for adult patients with laryngeal RMS^19–22^. However, considering that overall, only few cases of adult, localized, non-orbital, non-PM, head and neck RMS have been reported so far, it is unclear what effect chemotherapy exerts on the outcome of these patients. Future studies are encouraged to clarify the benefit and justify the application of different chemotherapy regimens to this patient group.

Evidence from the literature suggests that the chemosensitivity of adult RMS is similar to that of children, with overall-response rates between 74–89%^2,4,6,9,15,18,23^. Response to chemotherapy is a strong multivariate predictor in adult RMS and its impact on survival is independent of the timing or type of regimen used^9^. Little *et al*. reported significantly higher 5y-DMFS and LRFS rates among responders compared with non-responders (DMFS: 72%vs.19%,p = 0.004; LRFS: 77%vs.27%,p = 0.03)^18^.

We found a strong correlation between intermediate-risk disease and poor response to chemotherapy. This is similar to what has been observed in pediatric patients, which highlights the importance of considering the use of novel systemic agents for intermediate-risk patients. Several phase-II studies conducted by the Cooperative Oncology Group (COG) reported favorable efficacy and tolerability of various novel agents/combinations such as topotecan + cyclophosphamide, vincristine + irinotecan (VI), and irinotecan + carboplatin for intermediate-high risk cases^24–27^. In phase-III trials, however, the addition of topotecan to VAC did not improve the failure-free survival (FFS) or OS. Currently, the VAC/VI combination is under study in a COG phase-III trial and only preliminary results are available^28^.

Overall survival was significantly better for group-III patients who had delayed excisions (DPE) compared to those treated with definitive CRT. Furthermore, both local-control and overall survival of the group-III PR/NR cohort were significantly better, if their residual tumors were locally-controlled with combined SUR + XRT.

From the comparative analysis of the DPE vs. CRT group-III patients, it wasn’t possible to clearly infer what factors influenced authors’ decisions to perform DPE for their group-III patients. Patients with invasive tumors (T2) and/or tumors at unfavorable sites (e.g. PM) were equally likely to undergo DPE compared to those with non-invasive tumors (T1) and/or tumors at favorable sites. Nonetheless, radiographic-response to initial CT/CRT seemed to exert some influence in that >55% of DPE patients had radiographic-PR/NR compared to 16% of the CRT group. Kobayashi and colleagues investigated the local-control benefit of DPE following induction CT for 24 adults with group-III non-metastatic RMS of the head and neck. Their decision about DPE was based on primary tumor resectability in the initial imaging studies -information that could be retrieved for almost none of the patients in our dataset-. The extent of DPE (Radical vs. conservative resection), on the other hand, was based on the radiographic-response to induction CT on repeat imaging studies^29^. They found that DPE led to significantly better 3y-LRFS compared to definitive CRT, even within the patient group who achieved good radiographic response (CR/PR) to initial VAC^29^.

Whether DPE is necessary for group-III patients who achieved radiographic-CR is debatable. The COG evaluated the combination of DPE + XRT in 161 intermediate-risk group-III patients, 18 of whom achieved radiographic-CR before DPE. The study reported no correlation between radiographic-response and presence of viable tumor in the pathology specimens of DPE (p = 0.115)^30^. IRS (currently named COG) III-IV studies reported similar results for their DPE cohort, however, viable tumors were only present in 12% and 7% of group-III patients with radiographic-CR in IRS III and IV, respectively. Taken together, the IRS investigators recommended against performing DPE routinely for patients with radiographic-CR, since the great majority of them will have achieved a pathologic-CR^31^.

RMS in adults is not as radiosensitive as it is in children^18^. Consequently, recent evidence seems to support delayed surgery (DPE) over definitive CRT to improve local-control and OS of group-III patients. We believe the following elements should be thoroughly evaluated before deciding on DPE for group III patients:

(1) *Primary tumor resectability:* This typically varies with respect to surgical expertise and institutional capabilities, and is not solely dependent on tumor invasiveness or site; (2) *Extent of Nodal involvement*: In our dataset as well as Kobayashi analysis, patients without nodal involvement (N0) were much more likely to undergo DPE compared to their counterparts. (3) *Radiographic response to initial CT/CRT*: The satisfactory results -reported in this study and by others- on the strong predictive value of radiographic-CR for long-term local control, seems to contradict the necessity of delayed excisions for this patient group. Still, achieving pathologic-negative margins is a well-known strong independent predictor of long-term survival; (4) *Likelihood of achieving tumor-free margins and for surgical reconstruction to leave satisfactory functional and cosmetic results*: This is particularly important in considering DPE for head and neck tumors, where surgical resections can lead to unacceptable mutilation, as well as for PM regions where surgical resections often leads to incomplete resections owing to tumor inaccessibility. The long-term morbidity of combined DPE + XRT will be further evaluated in upcoming COG studies^30^.

One of the main questions of our study was whether or not XRT can be withheld for adult patients with non-PM group-I disease. In the current analysis, the addition of XRT didn’t add apparent local-control or OS benefit to group-I patients. Prior COG as well as European studies indicated that the only group-I patients who benefit from adjuvant XRT are those with non-embryonal histology, tumors at unfavorable sites or measuring >5 cm^11,32,33^. These features are well-established risk factors for local treatment failure in pediatric RMS. Thus, our results might simply reflect lower representation of these unfavorable features among our group-I cohort as opposed to a true lack of XRT benefit.

Our analysis demonstrated a significant univariate correlation between OS/PFS and non-embryonal histology, which is consistent with earlier studies^2,8,18,34^. Conversely, alveolar-histology was a non-significant predictor in the largest analysis of adult RMS so far^1^. We also found that IRS stage-II (unfavorable sites) was a significant multivariate predictor of poor local-control. Previous studies reported conflicting results; five retrospective adult series reported a greater risk of local recurrence in the unfavourable site group^5,7,8,18,34^, while others indicated lack of prognostic significance^1,4,9,14^.

While the prognostic significance of tumor site and histologic subtype in adults is controversial, the risk stratification system implemented by COG seems to be a strong predictive tool when applied to adult patients with non-metastatic disease^7,8^. Among our non-PM group-I cohort, the only significant difference between recurring and controlled tumors was in their risk group distribution. Consistent with our findings, age (>20 y.o) was a multivariate predictor of poor local-control in many adult series. Due to the risks of XRT-related morbidity, we believe postoperative-XRT can be withheld for select patients with negative surgical margins (R0), except for those with high risk for local recurrence, such as intermediate-risk disease, advanced IRS stage, large tumors or narrow surgical margins. Nonetheless, these aspects of our results, including the independent effect of age on the success of local-control, require further research.

## Conclusions and Implications for Future Research

There is a pressing need for the development of established treatment guidelines, including standardized chemotherapy regimens, for adults with non-metastatic RMS. Our findings highlight the local, distant and overall PFS benefit with the use of chemotherapy regimens, adapted from the pediatric RMS protocols, in adults with non-metastatic RMS. Development and assessment of novel chemotherapeutic agents is critical, especially for patients with intermediate-risk disease who could not benefit from conventional chemo-therapeutic regimens.

The local-control and OS of group-III patients seems to improve with intensified local therapy consisting of XRT + delayed SUR (DPE), especially within patient group who achieved radiographic-PR/NR to initial CT/CRT. It remains unclear which group III patients should be considered for DPE. We hope with our results to encourage future research to investigate the clinical as well as radiographic tumor features that can define potential candidacy for DPE.

The use of adjuvant XRT didn’t significantly affect the local-control or overall survival of non-PM group-I patients, yet the retrospective nature of the analysis and the lack of XRT guidelines makes it hard to reach definitive conclusions.

## Study Limitations

The following limitations should be considered in the interpretation of our findings: 1) The study’s retrospective nature; we were limited by the findings documented by other authors, 2) Deficient reporting on certain tumor and treatment related characteristics hindered our ability to conduct helpful subgroup analyses. Notable deficiencies were lack of data on exact chemotherapy doses and lack of information regarding the FOXO1 translocation status, which may explain the lack of prognostic significance of histology in the multivariate analysis. 3) In our dataset, young and middle-aged adult patients (<60 y.o) constituted more than 90% of all patients who received chemotherapy. Therefore, it is critical to indicate that the chemotherapy analysis results are more representative of patients <60 y.o. 4) Selective reporting bias. Despite the limitations raised by the retrospective nature of our study, retrospective analysis of large patient cohorts can be a reasonable alternative to randomized controlled trials in settings where the disease being studied is rare enough to deem prospective patient accrual impractical.

## Electronic supplementary material

Supplementary S1

Supplementary S2

Supplementary S3

Supplementary S4
