# Publisher Correction: Management of Low and Intermediate Risk Adult Rhabdomyosarcoma: A Pooled Survival Analysis of 553 Patients

**DOI:** 10.1038/s41598-018-29513-4

**Published:** 2018-07-25

**Authors:** Maha A. T. Elsebaie, Mohamed Amgad, Ahmed Elkashash, Ahmed Saber Elgebaly, Gehad Gamal E. l. Ashal, Emad Shash, Zeinab Elsayed

**Affiliations:** 1 Faculty of Medicine, Ain Shams University, Cairo, Egypt; 2 Department of Biomedical Informatics, Emory University School of Medicine, Atlanta, GA USA; 3 Kasr Al Ainy School of Medicine, Cairo University, Cairo, Egypt; 4 Faculty of Medicine, Al-Azhar University, Cairo, Egypt; 5 Medical Research Education and Practice Association (MREP), Cairo, Egypt; 6 Medical Oncology Department, National Cancer Institute, Cairo University, Cairo, Egypt; 7 Adult Sarcoma Division, Clinical Oncology Department, Ain Shams University Hospitals, Cairo, Egypt

Correction to: *Scientific Reports* 10.1038/s41598-018-27556-1, published online 19 June 2018

The supplementary file ‘Supplementary S2’ containing the full patient dataset was omitted from the original version of this Article. This has been corrected in the HTML version of the Article; the PDF version was correct at time of publication.

