# Supplementary material for: Management of Low and Intermediate Risk Adult Rhabdomyosarcoma: A Pooled Survival Analysis of 553 Patients
Source: Sci Rep. 2018 Jun 19;8:9337. doi: 10.1038/s41598-018-27556-1 (PMC6008292; doi:10.1038/s41598-018-27556-1)
Supplement: Supplementary file 1 — Supplementary S1 [file 41598_2018_27556_MOESM1_ESM.pdf]

Management of Low and Intermediate Risk Adult Rhabdomyosarcoma: A Pooled Survival Analysis of 553 Patients

Maha AT Elsebaie[1], Mohamed Amgad[†2], Ahmed Elkashash[†3], Ahmed Saber Elgebaly[4,5], Gehad Gamal El Ashal[3,5], Emad Shash[6], Zeinab Elsayed*[7]

1 Faculty of Medicine, Ain Shams University, Cairo, Egypt.

2 Department of Biomedical Informatics, Emory University School of Medicine, Atlanta, GA, USA.

3 Kasr Al Ainy School of Medicine, Cairo University, Cairo, Egypt

4 Faculty of Medicine, Al-Azhar University, Cairo, Egypt.

5 Medical Research Education and Practice Association (MREP)

6 Medical Oncology Department, National Cancer Institute, Cairo University, Cairo, Egypt.

7 Adult Sarcoma Division, Clinical Oncology Department, Ain Shams University Hospitals, Cairo, Egypt.

† Authors contributed equally

Reporting the Database search process

All the steps of the literature search were performed by two independent reviewers and any disagreements were resolved by discussion. **The full search strategies for each database were included.** The search strategies were copied and pasted exactly as run and included in full together with the line numbers for each search set.

Database	Date of search	From	Strategy (search saved as...)	Hits	Notes;
Medline/Pub-med	15 Jan 2015		**(**rhabdomyosarcoma OR myosarcoma OR soft tissue sarcoma/neoplasm OR soft tissue sarcomas OR rhabdomyosarcom* OR soft tissue sarcom* OR myosarcom***) AND (**adult OR adolescent OR aged OR aged, 80 and over OR age factors OR adult* OR adoles***) AND (**antineoplastic protocols OR antineoplastic combined chemotherapy protocols OR drug therapy, combination OR antineoplastic agents OR Antineoplastic Drug Combinations OR Chemoradiotherapy OR neoadjuvant therapy OR combined modality therapy OR combined modality treatment OR Drug resistance, multiple OR chemotherapy OR chemotherap* OR Multimodal* OR Therapy, Combined Modality**)**	21355	After duplications are removed within itself: **21347**
Cochrane (CENTRAL/CDSR)	15 Jan 2015		(rhabdomyosarcom* or soft tissue sarcom* or myosarcom* or rhabdomyosarcoma or myosarcoma or soft tissue sarcoma or soft tissue neoplasm or soft tissue sarcomas):ti,ab,kw and (age factors or aged or adult* or Adolescent) and (Chemotherap* or chemotherapy or Multimodal* or antineoplastic agents or antineoplastic combined drugs or antineoplastic combined treatment or antineoplastic combined chemotherapy protocols or antineoplastic agents or combined modality therapy or combined modality treatment or (antineoplastic agents and toxicity*) or drug resistance or Combined near therapy or Combined near drugs or Combined near modality):ti,ab,kw	**323**	The search filter "search all text" was used for adult set.

Medline/Ovid	16 Jan 2015		(Exp Rhabdomyosarcoma/ OR myosarcoma/ OR Exp soft tissue neoplasms/ OR sarcoma/ OR rhabdomyosarcom$.mp. OR rhabdomyosarcoma.mp. OR rhabdomyosar#om$.mp. OR sar#om$.mp. OR myosarcom$ OR soft tissue sarcoma.mp. OR soft tissue sarcomas.mp.) AND (Adult/ OR adolescent/ OR adult$.mp. OR adoles$.mp. OR aged/ OR age factors/ OR middle aged/ OR age distribution/) AND (exp Antineoplastic agents/ OR exp antineoplastic combined chemotherapy protocols/ OR exp Drug therapy, combination/ OR exp combined modality therapy/ OR rhabdomyosarcoma/dt, ra, su, tu, th [drug therapy, radiotherapy, surgery, therapeautic use, therapy] OR drug resistance, neoplasm/ OR dose response relationship, drug/ OR chemotherap$.mp. OR multi drugs.mp. OR multimodal$.mp.)	14654	1- after removal of duplicates within it (68): **14586**
CRD	19 Jan 2015			**4**	ti,ab search only
Scopus	19 Jan 2015		TITLE-ABS-KEY (rhabdomyosarcoma OR rhabdomyosarcom*) AND (adult OR adult* OR adolescent OR adoles*)	8941	1- 45 book chapter excluded.<br>2- 50 note excluded.<br>3- 2 book, 2 erratum excluded<br>4- After exclusion: **8842**<br>5- types: Article, review, conference paper, letter, short survey, editorial, article in press
ISI web of science	19 Jan 2015	1950-2015	(rhabdomyosarcoma OR rhabdomyosar?om*)) *AND* ((adult OR adoles*)	**574**	1- Topic field was chosen.<br>2- Medline database search/ books excluded: 574<br>3- Types: Meeting-article-case report- review – patent

EMBASE	3 Mar 2015	1980-2015	#1 **'rhabdomyosarcoma'**/exp<br>#2 rhabdomyosarcoma<br>#3 **rhabdomyosarcom***<br>#4 **rhabdomyosar?om***<br>#5 **'myosarcoma'**/de<br>#6 **myosarcom***<br>#7 **'soft tissue tumour'**/exp<br>#8 **soft** AND **tissue** AND **sarcoma**<br>#9 **soft** AND **tissue** AND **sarcomas**<br>#10 **'sarcoma'**/de<br>#11 **sar?om***<br>#12 #1 OR #2 OR #3 OR #4 OR #5 OR #6 OR #7 OR #8 OR #9 OR #10 OR #11<br>#13 **'adult'**/de<br>#14 **'adolescent'**/de<br>#15 **adult***<br>#16 **adoles***<br>#17 **'aged'**/de<br>#18 **'middle age'**/de<br>#19 #13 OR #14 OR #15 OR #16 OR #17 OR #18<br>#20 **'anti neoplastic agent'**/de<br>#21 **'drug combinations'**/de<br>#22 **'multimodality cancer therapy'**/de<br>#23**'chemotherapy'**/de<br>#24 **chemotherap***<br>#25 **multimodal***<br>**#26** #20 OR #21 OR #22 OR #23 OR #24 OR #25<br>#27 #12 AND #19 AND #26<br>#28 #27 AND [embase]/lim NOT [medline]/lim	**2890**	Duplicates not removed yet.
Updated Search Pubmed Only.	1 Sep 2016	20 Jan 2015- 1 Sep 2016	Rhabdomyosarcoma OR rhabdomyosarcom*	**687**	
All combined				Total= **49,249**	After removal of duplicates total number is= **36,063**<br><br>Duplicates= **13,186**

Head and Neck references

1. Jereb B, Haik BG, Ong R, Ghavimi F. Parameningeal rhabdomyosarcoma (including the orbit): results of orbital irradiation. International Journal of Radiation Oncology, Biology, Physics. 2057;11(12):2057-65.

2. Wu TT, Wang QY, Zhou SH, Zheng ZJ. Spindle cell rhabdomyosarcoma in the hypopharynx of an adult. International Journal of Clinical and Experimental Pathology. 2014;7(8):5254-8.

3. Russell JO, Revenaugh PC, Budd GT, Greskovich J, Scharpf J. Failed organ preservation strategy for adult laryngeal embryonal rhabdomyosarcoma. American journal of otolaryngology. 2014.

4. Neffendorf JE, Bagdonaite L, Mudhar HS, Pearson AR. Adult alveolar rhabdomyosarcoma of the lacrimal sac. Orbit. 2014;33(6):468-70.

5. Li JJ, Forstner D, Henderson C. Cutaneous pleomorphic rhabdomyosarcoma occurring on sun damaged skin: A case report. American Journal of Dermatopathology. 2014.

6. Hartmann S, Lessner G, Mentzel T, Kubler AC, Muller-Richter UD. An adult spindle cell rhabdomyosarcoma in the head and neck region with long-term survival: a case report. Journal of Medical Case Reports [Electronic Resource]. 2014;8(208).

7. Barosa J, Ribeiro J, Afonso L, Fernandes J, Monteiro E. Head and neck sarcoma: Analysis of 29 cases. European Annals of Otorhinolaryngology, Head and Neck Diseases. 2014;131(2):83-6.

8. Arul ASK, Verma S, Arul ASS, Verma R. Oral rhabdomyosarcoma-embryonal subtype in an adult: A rarity. Journal of Natural Science, Biology and Medicine. 2014;5(1):222-5.

9. Thompson CF, Kim BJ, Lai C, Grogan T, Elashoff D, St John MA, et al. Sinonasal rhabdomyosarcoma: prognostic factors and treatment outcomes. International Forum of Allergy & Rhinology. 2013;3(8):678-83.

10. Sahni P, Singhvi A, Nayak MT, Deora SS. Gingival Rhabdomyosarcoma in an Adult: A Unique Entity. Turk patoloji dergisi. 2013.

11. Robinson JC, Richardson MS, Neville BW, Day TA, Chi AC. Sclerosing rhabdomyosarcoma: report of a case arising in the head and neck of an adult and review of the literature. [Review]. Head and neck pathology. 2013;7(2):193-202.

12. Patil G, Halawar S, Sagari S, Babannavar R, Purohit S. Embryonal rhabdomyosarcoma occurring on mandibular gingiva in an adult. Journal of Clinical and Diagnostic Research. 2013;7(9):2088-9.

13. Palacios E, Quiroz-Casian A, Garcia LG, Daroca PJ, Neitzschman HR. Adult case of large sinonasal embryonal rhabdomyosarcoma with intracranial extension. Ear, Nose and Throat Journal. 2013;92(4):177-8.

14. Kelly A, Moran M, Primrose W. Alveolar ethmoidal rhabdomyosarcoma in a young adult male. BMJ Case Reports. 2013.

15. Siepermann M, Koscielniak E, Dantonello T, Klee D, Boos J, Krefeld B, et al. Oral low-dose chemotherapy: successful treatment of an alveolar rhabdomyosarcoma during pregnancy. Pediatric Blood & Cancer. 2012;58(1):104-6.

16. Vira D, Nguyen C, Mowry S. An unusual cause of hoarseness: Rhabdomyosarcoma of the larynx. Laryngoscope. 2011;121(SUPPL. 4).

17. Kukwa W, Wojtowicz P, Jagielska B, Sobczyk G, Kukwa A, Czarnecka AM. Laryngeal embryonal rhabdomyosarcoma in an adult - a case presentation in the eyes of geneticists and clinicians. BMC Cancer. 2011;11(166).

18. Kirkpatrick JP, Palta M, Riedel RF, Vredenburgh JJ, Cummings TJ, Green S, et al. Primary meningeal rhabdomyosarcoma. Sarcoma. 2011;2011.

19. Furdova A, Chynoransky M, Chorvath M, Svetlosakova Z. Malignant hemophtalmus as a first sign of orbital rhabdomyosarcoma in adult. Bratislava Medical Journal. 2011;112(12):715-6.

20. Fan YP, Chen SL, Liu XW, Gou XM, Xia WT. Laryngeal rhabdomyosarcoma: One case report and review of Chinese literature. American Journal of Case Reports. 2011;12:113-7.

21. Arya K, Vij H, Vij R, Rao NN. Rhabdomyosarcoma of mandible: A diagnostic predicament. Journal of Oral and Maxillofacial Pathology. 2011;15(3):320-5.

22. Li D-w, Xie J, Dong P. Alveolar Rhabdomyosarcoma of the Laryngohypopharynx: A Case Report. Clinical Oncology and Cancer Research. 2010;7(1):69-70.

23. Kragelund C, Meer S, Pallesen L, Reibel J. Clinico-pathologic conference: case 2. Embryonal rhabdomyosarcoma (RMS). Head and neck pathology. 2010;4(4):334-8.

24. Clement PMJ, Wagemans J, Beuselinck B, Nuyts S, Sciot R, Delaere P, et al. A case series of embryonal rhabdomyosarcoma of the head and neck in adults. Acta Clinica Belgica. 2010;65(6):404-10.

25. Papacharalampous GX, Manolopoulos L, Korres S, Dicoglou C, Bibas A. Adult laryngeal rhabdomyosarcoma: is aggressive treatment justified in all cases? A case report and review of the literature. [Review] [33 refs]. Journal of Laryngology & Otology. 2009;123(11).

26. Montone KT, Barr FG, Zhang PJ, Feldman MD, LiVolsi VA. Embryonal and alveolar rhabdomyosarcoma of parameningeal sites in adults: a report of 13 cases. International Journal of Surgical Pathology. 2009;17(1):22-30.

27. Matrka L, Cronin S, Agrawal A, Wakely Jr P. Head and neck rhabdomyosarcoma in the adult population. Laryngoscope. 2009;119(SUPPL.3).

28. Lamovec J, Volavsek M. Sclerosing rhabdomyosarcoma of the parotid gland in an adult. Annals of Diagnostic Pathology. 2009;13(5):334-8.

29. Nakagawa N, Tsuda T, Yamamoto M, Ito T, Futani H, Yamanishi K. Adult cutaneous alveolar rhabdomyosarcoma on the face diagnosed by the expression of PAX3-FKHR gene fusion transcripts. Journal of Dermatology. 2008;35(7):462-7.

30. Linden O, Greiff L, Wahlberg P, Vinge E, Kjellen E. Chemorefractory rhabdomyosarcoma treated with radiotherapy, bevacizumab, statins and surgery and maintenance with bevacizumab and chemotherapy. Onkologie. 2008;31(7):391-3.

31. Grebe HP, Steube D. Primary cerebral rhabdomyosarcoma presenting as haemorrhagic stroke. Zentralblatt fur Neurochirurgie. 2008;69(2):93-5.

32. Cil T, Altintas A, Isikdogan A. Rhabdomyosarcoma presenting with destructive large lesion of the face. Southern Medical Journal. 2008;101(1):104-5.

33. Shayah A, Agada FO, Karsai L, Stafford N. Adult laryngeal rhabdomyosarcoma: report of a case and literature review. [Review] [17 refs]. Annals of African Medicine. 2007;6(4):190-3.

34. Franca CM, Caran EM, Alves MT, Barreto AD, Lopes NN. Rhabdomyosarcoma of the oral tissues--two new cases and literature review. [Review] [15 refs]. Medicina Oral, Patologia Oral y Cirugia Bucal. 2006;11(2).

35. Kamiya E, Kitanishi T, Suzuki M, Sakurai H, Kosaki H, Shimizu T, et al. A case of rhabdomyosarcoma in the maxillary sinus. Practica Oto-Rhino-Laryngologica. 2005;98(6):471-5.

36. Huang YL, Tseng CF, Yang LK, Tsai CH. Rhabdomyosarcoma of the adult nasopharynx: A case report. Journal of Internal Medicine of Taiwan. 2005;16(3):146-50.

37. Dikbas O, Altundag K, Abali H, Turker A, Engin H, Sungur A, et al. Embryonal rhabdomyosarcoma of the larynx. Otolaryngology Head & Neck Surgery. 2005;133(1):160-2.

38. Maheshwari GK, Baboo HA, Gopal U, Wadhwa MK. Primary rhabdomyosarcoma of the larynx. Indian Journal of Otolaryngology and Head and Neck Surgery. 2004;56(2):138-41.

39. G.K. M, H.A. B, U. G, M.K. W. Primary rhabdomyosarcoma of the larynx. Indian Journal of Otolaryngology and Head and Neck Surgery. 2004;56:138-41.

40. Baldi A, Nicoletti G, Colella G, Di Marino MP, Persichetti P. Embryonal rhabdomyosarcoma of the tongue in old age. Oral Oncology. 2004;40(4):450-2.

41. Abali H, Aksoy S, Sungur A, Yalcin S. Laryngeal Involvement of Rhabdomyosarcoma in an Adult. World journal of surgical oncology. 2003;1:17.

42. Hu J, Liu S, Qiu J. Embryonal rhabdomyosarcoma of the middle ear. Otolaryngology - Head and Neck Surgery. 2002;126(6):690-2.

43. Bidwai SL, Srinivasan V. Rhabdomyosarcoma of the larynx in an adult. CME Bulletin Otorhinolaryngology Head and Neck Surgery. 2002;6(2):72-3.

44. De M, Banerjee A, Graham I, Dempster J. Alveolar rhabdomyosarcoma of the parotid gland. Journal of Laryngology & Otology. 2001;115(2):155-7.

45. Arita K, Sugiyama K, Tominaga A, Yamasaki F. Intrasellar rhabdomyosarcoma: case report. Neurosurgery. 2001;48(3):677-80.

46. Pandey M, Thomas G, Mathew A, Abraham EK, Somanathan T, Ramadas K, et al. Sarcoma of the oral and maxillofacial soft tissue in adults. European Journal of Surgical Oncology. 2000;26(2):145-8.

47. D.R. N, R. B, A.S. S, L. R. Alveolar rhabdomyosarcoma of paranasal sinus with cervical metastasis - A case report. Indian Journal of Otolaryngology and Head and Neck Surgery. 2000;52:162-5.

48. Della Libera D, Falconieri G, Zanella M. Embryonal "botryoid" rhabdomyosarcoma of the larynx: A clinicopathologic and immunohistochemical study of two cases. Annals of Diagnostic Pathology. 1999;3(6):341-9.

49. Ruske DR, Glassford N, Costello S, Stewart IA. Laryngeal rhabdomyosarcoma in adults. [Review] [17 refs]. Journal of Laryngology & Otology. 1998;112(7):670-2.

50. Jund R, Leunig A, Hagedorn H, Nerlich A, Grevers G. Embryonal rhabdomyosarcoma of the nose in an elderly patient, case report and review of the literature. [Review] [25 refs]. Auris, Nasus, Larynx. 1998;25(4):445-50.

51. Goto TK, Yoshiura K, Tanaka T, Kanda S, Ozeki S, Ohishi M, et al. A follow-up of rhabdomyosarcoma of the infratemporal fossa region in adults based on the magnetic resonance imaging findings: case reports. [Review] [20 refs]. Oral Surgery Oral Medicine Oral Pathology Oral Radiology & Endodontics. 1998;86(5):616-25.

52. Gorsky M, Epstein JB. Head and neck and intra-oral soft tissue sarcomas. Oral Oncology. 1998;34(4):292-6.

53. Akyol MU, Sozeri B, Kucukali T, Ogretmenoglu O. Laryngeal pleomorphic rhabdomyosarcoma. [Review] [15 refs]. European Archives of Oto Rhino Laryngology. 1998;255(6):307-10.

54. Pavithran K, Doval DC, Mukherjee G, Kannan V, Kumaraswamy SV, Bapsy PP. Rhabdomyosarcoma of the oral cavity: Report of eight cases. Acta Oncologica. 1997;36(8):819-21.

55. Ogawa I, Takata T, Nikai H, Suei Y, Ishikawa T. Alveolar rhabdomyosarcoma (solid variant) of the maxillary sinus in an adult. International Journal of Oral and Maxillofacial Surgery. 1996;25(2):122-3.

56. M.C. W, J.A. S, C.L. S, L. L. Orbital rhabdomyosarcoma fifty-seven years after radiotherapy for retinoblastoma. Orbit. 1996;15:97-100.

57. M. O, M. K, Y. A, I. Y. Lid swelling as the initial manifestation in a case of rhabdomyosarcoma in the orbit and paranasal sinus. Japanese Journal of Clinical Ophthalmology. 1996;50:1079-82.

58. Da Mosto MC, Rinaldo A, Marchiori C, Ferlito A. Laryngeal pleomorphic rhabdomyosarcoma: A critical review of the literature. Annals of Otology, Rhinology and Laryngology. 1996;105(4):289-94.

59. Colleoni M, Nelli P, Sgarbossa G, Pancheri F, Manente P. Primary cutaneous rhabdomyosarcoma in adults - Description of an uncommon aggressive disease. Acta Oncologica. 1996;35(4):494-5.

60. S. S, S. O, S. I, S. Y, H. I, H. S, et al. Perianal rhabdomyosarcoma: Report of a case. Journal of the Japan Society of Colo-Proctology. 1995;48:502-8.

61. Chen SY, Thakur A, Miller AS, Harwick RD. Rhabdomyosarcoma of the oral cavity. Report of four cases. Oral Surgery, Oral Medicine, Oral Pathology, Oral Radiology and. 1995;80(2):192-201.

62. Doval DC, Kannan V, Acharya RS, Mukherjee G, Shenoy AM, Bapsy PP. Rhabdomyosarcoma of the tongue. British Journal of Oral & Maxillofacial Surgery. 1994;32(3):183-6.

63. Freije JE, Gluckman JL, Biddinger PW, Wiot G. Muscle tumors in the parapharyngeal space. [Review] [23 refs]. Head & Neck. 1992;14(1):49-54.

64. Au E, Ang PT, Soh LT. Adult parameningeal rhabdomyosarcoma--a case report and literature review. [Review] [16 refs]. Singapore Medical Journal. 1992;33(4):415-7.

65. Peters E, Cohen M, Altini M, Murray J. Rhabdomyosarcoma of the oral and paraoral region. Cancer. 1989;63(5):963-6.

66. Sadeghi EM, Gingrass DJ, Surwillo EJ, Anderson T, Tang TT. Embryonal rhabdomyosarcoma. International Journal of Oral and Maxillofacial Surgery. 1988;17(3):198-200.

67. Renick B, Clark RM, Feldman L. Embryonal rhabdomyosarcoma: Presentation as a parotid gland mass. Oral Surgery, Oral Medicine, Oral Pathology. 1988;65(5):575-9.

68. Haerr RW, Turalba CIC, El-Mahdi AE, Brown KL. Alveolar rhabdomyosarcoma of the larynx: Case report and literature review. Laryngoscope. 1987;97(3 I):339-44.

69. Bras J, Batsakis JG, Lund MA. Rhabdomyosarcoma of the oral soft tissues. Oral Surgery, Oral Medicine, Oral Pathology. 1987;64(5):585-96.

70. Bradford R, Crockard HA, Isaacson PG. Primary rhabdomyosarcoma of the central nervous system: case report. Neurosurgery. 1985;17(1):101-4.

71. Diehn KW, Hyams VJ, Harris AE. Rhabdomyosarcoma of the larynx: a case report and review of the literature. Laryngoscope. 1984;94(2 Pt 1):201-5.

72. Chess J, Ni C, Yin RQ, Wang WJ, Cassady JR, Weichselbaum RR, et al. Rhabdomyosarcoma. International Ophthalmology Clinics. 1982;22(1):163-82.

73. Smith MT, Armbrustmacher VW, Violett TW. Diffuse meningeal rhabdomyosarcoma. Cancer. 1981;47(8):2081-6.

74. Kanegaonkar G, McDougall J, Grant HR. Rhabdomyosarcoma of the maxillary antrum in an adult. A case report with ultrastructural observations. Journal of Laryngology and Otology. 1981;95(8):863-72.

75. Pandhi SC, Mehra YN, Malik AK. Rhabdomyosarcoma of the head and neck. Journal of Laryngology & Otology. 1980;94(3):337-45.

76. Gorenstein A, Neel Iii HB, Weiland LH, Devine KD. Sarcomas of the larynx. Archives of Otolaryngology. 1980;106(1):8-12.

77. Makino Y. A clinicopathological study on soft tissue tumors of the head and neck. Acta Pathologica Japonica. 1979;29(3):389-408.

78. Winther LK, Lorentzen M. Rhabdomyosarcoma of the larynx. Report of two cases and a review of the literature. Journal of Laryngology and Otology. 1978;92(5):417-24.

79. Goepfert H, Lindberg RD, Sinkovics JG, Ayala AG. Soft tissue sarcoma of the head and neck after puberty. Treatment by surgery and postoperative radiation therapy. Archives of Otolaryngology. 1977;103(6):365-8.

80. Frugoni P, Ferlito A. Pleomorphic rhabdomyosarcoma of the larynx. A case report and review of the literature. Journal of Laryngology and Otology. 1976;90(7):687-98.

81. Hall-Jones J. Rhabdomyosarcoma of the larynx. Journal of Laryngology & Otology. 1975;89(9):969-76.

82. Deutsch M, Felder H. Rhabdomyosarcoma of the ear-mastoid. Laryngoscope. 1974;84(4):586-92.

83. Olurin O. Orbital rhabdomyosarcoma in pregnancy. Cancer. 1969;24(5):1013-6.

84. Kaloyannides TM. Pleomorphic rhabdomyosarcoma of the gingiva. Report of a case. Oral Surgery, Oral Medicine, Oral Pathology. 1969;27(2):150-5.

85. Masson JK, Soule EH. Embryonal rhabdomyosarcoma of the head and neck. Report on eighty-eight cases. The American Journal of Surgery. 1965;110(4):585-91.

86. Ashton N, Morgan G. Embryonal sarcoma and embryonal rhabdomyosarcoma of the orbit. Journal of Clinical Pathology. 1965;18(6):699-714.

87. Wiss K, Solomon AR, Raimer SS, Lobe TE, Gourley W, Headington JT. Rhabdomyosarcoma presenting as a cutaneous nodule. Archives of Dermatology. 1687;124(11):1687-90.

88. Haik BG, Jereb B, Smith ME, Ellsworth RM, McCormick B. Radiation and chemotherapy of parameningeal rhabdomyosarcoma involving the orbit. Ophthalmology. 1001;93(8):1001-9.

89. Inglis H, Ng WY, Ghabrial R. Adult ocular adnexal rhabdomyosarcoma presenting with inferior fornix lesion without proptosis. Clinical and Experimental Ophthalmology.42(7):688-9.

Genitourinary references

1. Haga K, Kashiwagi A, Nagamori S, Yamashiro K. Adult paratesticular rhabdomyosarcoma. Nature Clinical Practice Urology. 2005;2(8):398-402.

2. Rao CR, Srinivasulu M, Naresh KN, Doval DC, Hazarika D. Adult paratesticular sarcomas: A report of eight cases. Journal of Surgical Oncology. 1994;56(2):89-93.

3. Sexton WJ, Lance RE, Reyes AO, Pisters PWT, Tu SM, Pisters LL. Adult prostate sarcoma: The M. D. Anderson cancer center experience. Journal of Urology. 2001;166(2):521-5.

4. Musser JE, Assel M, Mashni JW, Sjoberg DD, Russo P. Adult prostate sarcoma: The memorial sloan kettering experience. Urology. 2014;84(3):624-8.

5. Palmer MA, Viswanath S, Desmond AD. Adult prostatic rhabdomyosarcoma. British Journal of Urology. 1993;71(4):489-90.

6. Tang HW, Lin T, Zeng H, Wang XD. Alveolar rhabdomyosarcoma of the tunica vaginalis presenting as a tender hydrocele. Kaohsiung Journal of Medical Sciences. 2013;29(10):584-5.

7. Asahina M, Saito T, Arakawa A, Suehara Y, Takagi T, Hisasue SI, et al. A case of primary spindle cell variant of embryonal rhabdomyosarcoma of the prostate. International Journal of Clinical and Experimental Pathology. 2014;7(8):5181-5.

8. Erbay ME, Tarhan F, Barişik NÖ, Kuyumcuoğlu U. A case of testicular rhabdomyosarcoma. International Urology and Nephrology. 2004;36(1):73-5.

9. Hoekstra HJ, Wobbes T, Brouwers TM, Postma A, Schraffordt Koops H. Embryonal rhabdomyosarcoma of spermatic cord. Urology. 1980;16(4):360-3.

10. Keenan DJM, Graham WH, Hayes D. Embryonal rhabdomyosarcoma of the prostatic-urethral region in an adult. British Journal of Urology. 1985;57(2):241.

11. Li D, Li Y, Wang K, Li H, Tang Y, Wei X, et al. Embryonal rhabdomyosarcoma of the tunica dartos in the scrotum. Journal of Ultrasound in Medicine. 2011;30(1):105-9.

12. Punekar SV, Sane SY, Patel B, Pardanani DS. Embryonal rhabdomyosarcoma of urinary bladder in an adult. Journal of Postgraduate Medicine. 1982;28(3):174-6.

13. Bouchikhi AA, Mellas S, Tazi MF, Lahlaidi K, Kharbach Y, Benhayoune K, et al. Embryonic paratesticular rhabdomyosarcoma: a case report. Journal of Medical Case Reports [Electronic Resource]. 2013;7(93).

14. Khandelwal A, Gupta SK, Singh M, Kumar V, Tiwari R, Singh A. High-grade pleomorphic sarcoma of the scrotum: A rare clinical entity. UroToday International Journal. 2012;5(2).

15. Sencan A, Mir E, Sencan AB, Ortac R. Intrascrotal paratesticular rhabdomyoma: A case report. Acta Paediatrica, International Journal of Paediatrics. 2000;89(8):1020-2.

16. Olney LE, Narayana A, Loening S, Culp DA. Intrascrotal rhabdomyosarcoma. Urology. 1979;14:113-25.

17. Sugie S, Tsukino H, Mukai S, Nakahara K, Kamoto T. Intrascrotal rhabdomyosarcoma: A report of two cases. Nishinihon Journal of Urology. 2013;75(2):85-9.

18. Hermans BP. Is retroperitoneal lymph node dissection necessary for adult paratesticular rhabdomyosarcoma? Journal of Urology. 1998;160(6 I):2074-7.

19. Spunt SL, Sweeney TA, Hudson MM, Billups CA, Krasin MJ, Hester AL. Late effects of pelvic rhabdomyosarcoma and its treatment in female survivors. Journal of Clinical Oncology. 7143;23(28):7143-51.

20. Dang ND, Dang PT, Samuelian J, Paulino AC. Lymph node management in patients with paratesticular rhabdomyosarcoma: A Population-Based Analysis. Cancer. 2013;119(17):3228-33.

21. Arlen M, Grabstald H, Whitmore Jr WF. Malignant tumors of the spermatic cord. Cancer. 1969;23(3):525-32.

22. Hamilton CR, Pinkerton R, Horwich A. The management of paratesticular rhabdomyosarcoma. Clinical Radiology. 1989;40(3):314-7.

23. Lee HY, Tsai CC, Huang CH, Li WM, Yeh HC, Wu WJ, et al. Mixed-type paratesticular rhabdomyosarcoma--a case report. Kaohsiung Journal of Medical Sciences. 2011;27(6):239-41.

24. Jacobellis U, Ricco R. Myosarcoma of the urinary bladder in adult patients. Urologia. 1981;48(4):734-8.

25. Mackenzie AR, Whitmore Jr WF, Melamed MR. Myosarcomas of the bladder and prostate. Cancer. 1968;22(4):833-44.

26. Onuora VC, Akumabor PN, Aghahowa JA. Non-germ cell testicular tumours in Nigerians. Tropical and Geographical Medicine. 1989;41(4):358-60.

27. Tsubura A, Shikata N, Morii S, Kajimoto M, Oohara T. An operative case of spermatic cord rhabdomyosarcoma--with a review of similar cases in Japan. Gan no rinsho Japan journal of cancer clinics. 1985;31(13):1750-5.

28. Matveev B, Guraryi L, Volkova M, Matveev V. Outcome analysis of 72 cases of paratesticular rhabdomyosarcoma. European Urology Supplements. 2002;1(1):95-.

29. Manzione A, de Garcia VC, Graciotti Júnior O, Mallamann A, Camargo JC. Pararectal rhabdomyosarcoma. Report of 2 cases. Revista do Hospital das Clinicas de Faculdade de Medicina da Universidade de Sao Paulo. 1984;39(5):243-7.

30. Sasajima K, Okawa K, Sasamoto Y, Shirota A, Aihara K. Pararectal rhabdomyosarcoma: report of a case. Diseases of the Colon & Rectum. 1980;23(8):576-7.

31. 박관규, 원종진, 김주남, Song J. Paratesticlular Rhabdomyosarcoma in an Adult. Korean Journal of Urology. 2002;43(10):901-12.

32. Li L, Wang Y. Paratesticular embryonal rhabdomyosarcoma-- a case report. Zhonghua bing li xue za zhi Chinese journal of pathology. 2004;33(3):259.

33. Iazmal'ian SO, Fridman GB. [Paratesticular embryonal rhabdomyosarcoma]. [Russian]. Urologiia i Nefrologiia. 1972;37(4):66-7.

34. Toro M, Gomez-Bermudo F, Gomez-Perez F, Valverde A, Morales C, Nogales F. Paratesticular embryonary rhabdomyosarcomas. Actas Urologicas Espanolas. 1981;5(6):389-92.

35. Demir A, Önol FF, Türkeri L. Paratesticular pleomorphic rhabdomyosarcoma in an adult. International Urology and Nephrology. 2004;36(4):577-8.

36. Busato Jr WFS, Bettega LBP, Pereira E, Oliveira R, Soldatelli DCS. Paratesticular pleomorphic rhabdomyosarcoma in an elderly man. Chirurgia. 2009;22(1):49-50.

37. Gómez Velázquez M, Ortega Bevia MC, Asuero Mantero M, Leal Arenas J. Paratesticular rhabdomyosarcoma. Actas Urologicas Espanolas. 1991;15(3):266-9.

38. Gutiérrez-Vilches CE, De Rubens-Villalvazo J. Paratesticular rhabdomyosarcoma. Boletin medico del Hospital Infantil de Mexico. 1974;31(1):123-7.

39. Kage M, Kojiro M, Arakawa M, Nakamura Y, Kawada H. Paratesticular rhabdomyosarcoma. Acta Pathologica Japonica. 1983;33(4):817-21.

40. M.C. U, A.I. A, L. K, E. G, Y. G, D. E. Paratesticular rhabdomyosarcoma. Turkish Journal of Cancer. 1998;28:80-3.

41. Sant GR, Raju GC, Jankey N. Paratesticular rhabdomyosarcoma. West Indian Medical Journal. 1979;28(4):246-50.

42. Sinclair-Smith CC. Paratesticular rhabdomyosarcoma. South African Journal of Surgery. 1972;10(1):56-7.

43. Ketiku KK, Esho JO, Azodo MVU. Paratesticular rhabdomyosarcoma in adolescents. European Urology. 1988;14(3):245-8.

44. Kattan J, Culine S, Terrier-Lacombe MJ, Theodore C, Droz JP. Paratesticular rhabdomyosarcoma in adult patients: 16-year experience at Institut Gustave-Roussy. [Review] [18 refs]. Annals of Oncology. 1993;4(10):871-5.

45. Kaneti J, Inbar IJ, Sober I, Smailowitz Z. Paratesticular rhabdomyosarcoma in an adult. Urology. 1980;16(6):614-6.

46. Kumar R, Kapoor R, Khosla D, Kumar N, Ghoshal S, Mandal AK, et al. Paratesticular rhabdomyosarcoma in young adults: A tertiary care institute experience. Indian Journal of Urology. 2013;29(2):110-3.

47. Livne P, Nseyo U, Wolf RM, Huben RP, Pontes JE. PARATESTICULAR RHABDOMYOSARCOMA ROSWELL PARK MEMORIAL INSTITUTE NEW-YORK USA EXPERIENCE. Journal of Urology. 1985;133(4 PART 2):148A-A.

48. De Castro Barbosa F, Rosell Costa D, Aguera Fernandez LG, Isa Kroon W, Sanchez de la Muela P, Robles Garcia JE, et al. [Paratesticular rhabdomyosarcoma with intracaval neoplastic involvement: presentation of a case and review of the literature]. Actas urologicas espanolas. 1991;15:469-72.

49. Rosas-Uribe A, Luna MA, Guinn GA. Paratesticular rhabdomyosarcoma. A clinicopathologic study of seven cases. American Journal of Surgery. 1970;120(6):787-91.

50. Knight B, Tiltman AJ. Paratesticular rhabdomyosarcoma. Case reports. South African Medical Journal. 1986;69(11):702-4.

51. Perez Herms S, Castellanos Acosta R, Cortadellas Angel R, Guzman Fernandez A, Ballesteros Sampol JJ. [Paratesticular rhabdomyosarcoma. Report of 2 cases]. Actas urologicas espanolas. 1991;15:491-4.

52. Kasmaoui E, Jira H, Alami M, Ghadouane M, Ameur A, Abbar M. [Paratesticular rhabdomyosarcoma. Three case reports]. [French]. Annales d Urologie. 2001;35(5):296-300.

53. Littmann R, Tessler AN, Valensi Q. Paratesticular rhabdomyosarcoma: a case presentation and review of the literature. Journal of Urology. 1972;108(2):290-2.

54. Navarro TP, Prosper AV, Gomez JP, Ebri MLP, Domenech RL, Garcia JMO, et al. Paratesticular rhabdomyosarcoma: A case report. Archivos Espanoles de Urologia. 2013;66(3):305-7.

55. Pastor Navarro T, Verges Prosper A, Planelles Gomez J, Perez Ebri ML, Llorente Domenech R, Osca Garcia JM, et al. Paratesticular rhabdomyosarcoma: A case report. Archivos Espanoles de Urologia. 2013;66(3):305-7.

56. Garmendía Larrea JC, Arrinda Heregy JM, Rodríguez Andrés JA, Campa Bortolo JM, Cátedra García A. Paratesticular rhabdomyosarcoma: report of a case. Archivos Espanoles de Urologia. 1993;46(2):139-40.

57. Blyth B, Mandell J, Bauer SB, Colodny AH, Grier HE, Weinstein HJ, et al. Paratesticular rhabdomyosarcoma: results of therapy in 18 cases. The Journal of urology. 1990;144:1450-3.

58. Nesa S, Lefebvre Y, Montfort JL, Wese FX, Van Cangh P. [Paratesticular rhabdomyosarcoma]. [French]. Acta Urologica Belgica. 1994;62(3):37-42.

59. Mamveev BP, Gurarii LL, Volkova MI, Matveev VB, Khalaf'ian EA. [Paratesticular rhabdomyosarcoma]. [Russian]. Urologiia. 2003;2:18-21.

60. Martin-Laborda y Bergasa F, Virseda Chamorro M, Vallejo Herrador J, Vallejo Ocana C, Colmenarejo Rubio A, Moreno Muro M. [Paratesticular rhabdomyosarcoma]. [Spanish]. Actas Urologicas Espanolas. 1992;16(4):346-50.

61. Kabiri H, Elmansari O, al Bouzidi A, Taberkant M, Benomar S, Draoui D. [Paratesticular rhabdomyosarcomas. Apropos of a case]. [French]. Journal d Urologie. 1996;102(4):176-9.

62. Bergerot P, Dilhuydy JM, Bui NB. Paratesticular sarcomas (concerning 4 cases and a review of literature). Bordeaux Medical. 1981;14(20):1385-8.

63. Bissada NK, Finkbeiner AE, Redman JF. Paratesticular sarcomas: review of management. Journal of Urology. 1976;116(2):198-200.

64. Nakamura T, Endo H, Otsuka K, Murayama N, Mizumachi Y. Paratesticular sarcomas: Two case reports. Nishinihon Journal of Urology. 1993;55(2):249-51.

65. Folpe AL, Weiss SW. Paratesticular soft tissue neoplasms. Seminars in Diagnostic Pathology. 2000;17(4):307-18.

66. Williams G, Banerjee R. Paratesticular tumours. British Journal of Urology. 1969;41(3):332-9.

67. Gowing NF, Morgan AD. PARATESTICULAR TUMOURS OF CONNECTIVE TISSUE AND MUSCLE. British journal of urology. 1964;36:SUPPL:78-84.

68. Ziari M, Sonpavde G, Shen S, Zhai J, Teh BS, Lerner SP. Patients with unusual bladder malignancies and a rare cause of splenomegaly: Case 2. Rhabdomyosarcoma of the urinary bladder in an adult. Journal of Clinical Oncology. 2005;23(19):4459-60.

69. Casey DL, Wexler LH, LaQuaglia MP, Meyers PA, Wolden SL. Patterns of failure for rhabdomyosarcoma of the perineal and perianal region. International journal of radiation oncology, biology, physics. 2014;89:82-7.

70. Nseyo UO, Livne PM, Wolf RM, Pontes JE, Huben RP. Pelvic rhabdomyosarcoma: a review of the RPMI experience. Urology. 1986;28(6):456-61.

71. Serrano O A, Murillo C Á, Alcalá M I, Alcántara A, De La Cruz R, Galeazzi V, et al. Perianal rhabdomyosarcoma. Medicina Interna de Mexico. 2001;17(1):39-42.

72. Saito S, Oki S, Ishihara S, Yamaguchi S, Ike H, Shimada H, et al. Perianal rhabdomyosarcoma: Report of a case. Journal of the Japan Society of Colo-Proctology. 1995;48(6):502-8.

73. Goble M, Clarke T, Durrani A, Teasdale C. Pleomorphic rhabdomyosarcoma of the urinary bladder in association with recurrent urinary-tract infection. European Journal of Surgical Oncology. 1989;15(2):155-7.

74. Liu ZW, Zhang XQ, Hou GL, Zhang ZL, Qin ZK, Han H, et al. Primary adult intratesticular rhabdomyosarcoma: results of the treatment of six cases. International Journal of Urology. 2011;18(2):171-4.

75. Kobayashi M, Kobayashi O, Suzuki H. Primary bladder sarcoma. Three case reports and review of the Japanese literature. Japanese Journal of Urology. 1983;74(1):111-24.

76. Nabi G, Dinda AK, Dogra PN. Primary embryonal rhabdomyosarcoma of prostate in adults: diagnosis and management. [Review] [20 refs]. International Urology & Nephrology. 2002;34(4):531-4.

77. Tochigi T, Itoh S, Kawamura S, Satoh M, Hayashi T, Sakagami Y, et al. Primary embryonal rhabdomyosarcoma of the bladder in an adult female. A case diagnosed by electron microscope and immunohistochemical methods. Nishinihon Journal of Urology. 1988;50(4):1301-6.

78. Fang S, Sun Y, Wang MPHY. Primary embryonal rhabdomyosarcoma of the kidney in an adult: A case report. International Journal of Radiation Research. 2014;12(2):199-202.

79. Bisceglia M, Magro G, Carosi I, Cannazza V, Ben Dor D. Primary embryonal rhabdomyosarcoma of the prostate in adults: report of a case and review of the literature. [Review]. International Journal of Surgical Pathology. 2011;19(6):831-7.

80. Yushita Y, Matsuya F, Yamashita S, Hayashi M, Yura M, Sakuragi T, et al. PRIMARY EMBRYONAL TYPE RHABDOMYOSARCOMA OF THE BLADDER A CASE REPORT CONCERNING AN ADULT. Nishinihon Journal of Urology. 1986;48(2):505-9.

81. Romero Tenorio M, Fariñas Varo JM, Báez Perea JM, Almaised J, Ramírez Chamorro R, Parra Martínez JL. Primary embryonic rhabdomyosarcoma of the prostate. An unusual location. Archivos Espanoles de Urologia. 1989;42(6):577-9.

82. Heitzman J, Rao P. Primary Paratesticular Rhabdomyosarcoma: A Clinicopathologic Study of 25 Cases. Laboratory Investigation. 2013;93(Suppl. 1):216A-A.

83. Wang CJ, Li J, Qin J. Primary pleomorphic rhabdomyosarcoma of the adrenal gland in an adult: A case report. Oncology Letters. 2014;7(1):137-9.

84. Mainguene C, Choquenet C, Cucchi JM, Dupre F, Monticelli I, Michiels JF, et al. [Primary pleomorphic rhabdomyosarcoma of the kidney in adults: unusual tumor]. [French]. Progres en Urologie. 2003;13(4):679-82.

85. Gürel D, Tuna B, Yörükoğlu K, Aslan G. Primary renal rhabdomyosarcoma: A case report. Turk Patoloji Dergisi. 2015;31(1):56-9.

86. Nakagawa Y, Komatsu Y, Okamure A. PRIMARY RHABDOMYOSARCOMA OF THE BLADDER A CASE REPORT CONCERNING AN ADULT AND REVIEW OF THE JAPANESE LITERATURE. Rinsho Hinyokika. 1991;45(7):511-3.

87. Ohsawa S, Shimizu H, Tsuboi N, Oki M, Akimoto M. Primary rhabdomyosarcoma of the bladder in an adult woman. Japanese Journal of Clinical Urology. 1993;47(8):583-6.

88. Hakozaki M, Hojo H, Kuze T, Tajino T, Yamada H, Kikuta A, et al. Primary rhabdomyosarcoma of the sacrum: a case report and review of the literature. [Review] [23 refs]. Skeletal Radiology. 2008;37(7):683-7.

89. Hashine K, Akiyama M, Inoue Y, Akiyama K, Sumiyoshi Y, Mandai K, et al. Primary sarcoma of the bladder: Three case reports. Nishinihon Journal of Urology. 1994;56(5):565-9.

90. Rodriguez D, Barrisford GW, Sanchez A, Preston MA, Kreydin EI, Olumi AF. Primary spermatic cord tumors: disease characteristics, prognostic factors, and treatment outcomes. Urologic Oncology. 2014;32(1).

91. Dall'Igna P, Bisogno G, Ferrari A, Treuner J, Carli M, Zanetti I, et al. Primary transcrotal excision for paratesticular rhabdomyosarcoma: Is hemiscrotectomy really mandatory? Cancer. 2003;97(8):1981-4.

92. Marberger Jr M, Sauer H, Mikuz G. Prognosis of paratesticular sarcoma: three case reports. Zeitschrift fur Urologie und Nephrologie. 1975;68(7):473-8.

93. La Quaglia MP, Ghavimi F, Herr H, Mandell L, Pennenberg D, Hajdu S, et al. Prognostic factors in bladder and bladder-prostate rhabdomyosarcoma. Journal of Pediatric Surgery. 1066;25(10):1066-72.

94. P. C, M. G, N. DA, T. P, E. D, C. I. Prostate embryonal rhabdomyosarcoma in adults: Case report and review of literature. Reports of Practical Oncology and Radiotherapy. 2013;18:310-5.

95. Beyzadeoglu M, Balkan M, Ozgok Y, Demiriz M, Pak Y. Prostate rhabdomyosarcoma in a young adult: a case study. Radiation Medicine. 1997;15(3):199-201.

96. Musquera Felip M, de Rodriguez Ledesma JM, Cetina Herrando A, Salvador Bayarri J, Villavicencio Mavrich H. [Prostate rhabdomyosarcoma]. [Spanish]. Actas Urologicas Espanolas. 2004;28(10):Nov-Dec.

97. M. X, Y.-K. Z, L. Z. Prostate sarcoma (A report of 11 cases). Fudan University Journal of Medical Sciences. 2003;30:84-6.

98. Chen HJ, Xu M, Zhang L, Zhang YK, Wang GM. [Prostate sarcoma: a report of 14 cases]. [Chinese]. Zhong Hua Nan Ke Xue. 2005;11(9):683-5.

99. Lu D, Tian J, Li M. Prostate sarcoma: A report of six cases. Chinese Journal of Clinical Oncology. 2005;32(12):712-4.

100. Vesga Molina F, Albisu Tristan A, Blasco de Villalonga M, Llarena Ibarguren R, Pertusa Pena C. [Prostate-bladder rhabdomyosarcoma: therapeutic approaches]. Archivos espanoles de urologia. 1994;47:985-9.

101. Waring PM, Newland RC. Prostatic embryonal rhabdomyosarcoma in adults. A clinicopathologic review. [Review] [48 refs]. Cancer. 1992;69(3):755-62.

102. Munoz Velez D, Rebassa Llull M, Anton Valenti E, Ramos Asensio R, Riera Mari V, Gutierrez Sanz-Gadea C, et al. [Prostatic rhabdomyosarcoma in adults]. [Review] [21 refs] [Spanish]. Archivos Espanoles de Urologia. 1998;51(9):883-8.

103. d'Udekem F, Van Cangh PJ, Hennebert P. Prostatic sarcoma. Acta Urologica Belgica. 1980;48(1):56-64.

104. Dargent F, Colombeau P, Paraf F, Dumas JP, Paulhac P. Prostatic sarcoma: Report of two cases. Progres en Urologie. 2006;16(5):613-6.

105. Mechtersheimer G, Haas R, Katus HA, Moller P. Pseudoleukaemic pancytopenia as presenting symptom in alveolar rhabdomyosarcoma of testis. Pathologe. 1989;10(4):252-6.

106. Meir K, Wygoda M, Reichman O, Gofrit ON, Pizov G. Puerperal renal rhabdomyosarcoma: case report and review of the literature. Urologic Oncology. 2006;24(1):40-3.

107. Rigatti P, Apicella F, Belvisi P, Lania C. A rare case of rhabdomyosarcoma of the bladder in an adult patient. Minerva Urologica. 1981;33(3):185-90.

108. Mazerolles C, Chevreau C. Rare malignant tumours of the prostate. Bulletin du Cancer. 2007;94(6 SUPPL.):F44-F9.

109. Zanoni F, Pizzocaro G. Report of 5 cases of paratesticular rhabdomyosarcoma. Tumori. 1975;61(3):255-9.

110. E. K, O. S, F. E, F. O, T. E, M. T. Retrospective analysis of sarcomas of the urogenital system. Turk Uroloji Dergisi. 2005;31:49-54.

111. Peterson LJ, Paulson DF. Rhabdomyosarcoma in adult prostate. Urology. 1974;3(6):689-92.

112. Dupree WB, Fisher C. Rhabdomyosarcoma of prostate in adult. Long-term survival and problem of histologic diagnosis. Urology. 1982;19(1):80-2.

113. P. P, R. S, T. S, G. N. Rhabdomyosarcoma of prostate presenting as bladder outlet obstruction in a young adult. ecancermedicalscience. 2013;7.

114. Dikranian HA. Rhabdomyosarcoma of spermatic cord. Urology. 1977;9(3):329-31.

115. Sago AL, Novicki DE. Rhabdomyosarcoma of spermatic cord. Urology. 1982;19(6):606-8.

116. Wróbel S, Steinmetz L. Rhabdomyosarcoma of spermatic cord. Polski Przeglad Chirurgiczny. 1976;48(2 A):273-5.

117. Boujnah H, Abid I, El Mazni F, Ben Jilani S, Zmerli S. Rhabdomyosarcoma of spermatic cord (about one case of review literature). Tunisie Medicale. 1989;67(6-7):439-42.

118. Prabhu R, Natarajan A, Shenoy R, Vaidya K. Rhabdomyosarcoma of spermatic cord in a 65-year-old man presenting as a groin swelling. BMJ Case Reports. 2013.

119. Kerr KM, Grigor KM, Tolley DA. Rhabdomyosarcoma of the adult urinary bladder after radiotherapy for carcinoma. Clinical Oncology. 1989;1(2):115-6.

120. Musierowicz A, Liebhardt M, Bienias B, Kubicz Z, Ryszkiewicz B. [Rhabdomyosarcoma of the bladder in a 70-year-old man]. [Polish]. Wiadomosci Lekarskie. 1807;37(22):1807-10.

121. Evans AT, Bell TE. Rhabdomyosarcoma of the bladder in adult patients: report of three cases. Journal of Urology. 1965;94(5):573-5.

122. Lozano Ortega JL, Mayayo Artal E, Rey Ramos A. Rhabdomyosarcoma of the bladder in adults. Actas Urologicas Espanolas. 1985;9(4):365-8.

123. Tamarit L, Tramoyères Sr A, Llopis B, Tramoyères Jr A. Rhabdomyosarcoma of the bladder in adults. Journal d''urologie et de nephrologie. 1971;77:Suppl:546-51.

124. Yasui T, Tanaka H, Sasaki S, Kohri K. Rhabdomyosarcoma of the bladder in an adult. Urologia Internationalis. 1999;63(2):144-6.

125. Martini E, Rosati A, Sindici G. Rhabdomyosarcoma of the bladder in the adult. Minerva Urologica. 1979;31(2):133-6.

126. Solivetti FM, D'Ascenzo R, Molisso A, Calugi V, Rossi P. Rhabdomyosarcoma of the funiculus. Journal of Clinical Ultrasound. 1989;17(7):521-2.

127. Egorov VP. Rhabdomyosarcoma of the kidney (1 case). Voprosy Onkologii. 1977;23(12):84-6.

128. Hiura M, Hayashi T, Taki Y, Ikai K, Ryoji O, Kiriyama T. [Rhabdomyosarcoma of the kidney: report of a case]. [Review] [40 refs] [Japanese]. Hinyokika Kiyo Acta Urologica Japonica. 1404;33(9):1404-10.

129. Painter MR, O'Shaughnessy EJ, Larson PH, Ribbe RE. Rhabdomyosarcoma of the male urethra. Journal of Urology. 1968;99(4):455-7.

130. Stanton MJ. Rhabdomyosarcoma of the paratesticular tissues. Report of a case. New Zealand Medical Journal. 1966;65(406):389-91.

131. Venkov G, Khristova S, Mavrov K. [Rhabdomyosarcoma of the penis]. [Bulgarian]. Khirurgiia. 2006;6:59-61.

132. Saussine C, Jacqmin D, Babin-Boilletot A, Ghnassia JP, Bollack C. [Rhabdomyosarcoma of the prostate. Diagnostic course and current therapy. Report of a case of a 17-year-old boy]. [French]. Progres en Urologie. 1992;2(5):913-8.

133. Seino K, Tanji S, Yamamoto T, Fujizuka I, Ohinata M, Kubo T, et al. [Rhabdomyosarcoma of the prostate: report of a case and review of the literature]. Hinyokika kiyo Acta urologica Japonica. 1987;33:1906-12.

134. Ito H, Murase T, Takashi M, Sobazima T, Miyake K, Mitsuya H. [Rhabdomyosarcoma of the prostate]. [Japanese]. Hinyokika Kiyo Acta Urologica Japonica. 1986;32(1):119-23.

135. Kao HW, Wu CJ, Cheng MF, Lee SS, Chen CY. Rhabdomyosarcoma of the renal pelvis. Journal of Medical Sciences. 2005;25(4):207-10.

136. Hays DM, Mirabal VQ, Patel HR, Shore N, Woolley MM. Rhabdomyosarcoma of the spermatic cord. Surgery. 1969;65(5):845-9.

137. Ninfo V. Rhabdomyosarcoma of the spermatic cord. Rivista di Anatomia Patologica e di Oncologia. 1967;30(2):314-29.

138. Tanimura H, Furuta M. Rhabdomyosarcoma of the spermatic cord. Cancer. 1968;22(6):1215-20.

139. Zegel HG, Goldsmith HS. Rhabdomyosarcoma of the spermatic cord. Clinical Oncology. 1976;2(3):261-6.

140. Brenez J, Rettmann R. Rhabdomyosarcoma of the spermatic cord. Report of a case and review of the literature. Acta Urologica Belgica. 1973;41(4):609-20.

141. Hoyos Fitto C, Isusquiza Carro JI, Pertusa Pena C, Perez-Castro Ellendt E. [Rhabdomyosarcoma of the spermatic cord: apropos of a case]. [Spanish]. Archivos Espanoles de Urologia. 1979;32(3):255-60.

142. Skeel DA, Drinker Jr HR, Witherington R. Rhabdomyosarcoma of the spermatic cord: report of 3 cases with review of the literature. Journal of Urology. 1975;113(2):279-84.

143. Kostakopoulos A, Delakas D, Deliveliotis C, Dimopoulos MA, Sofras F. Rhabdomyosarcoma of the testis. Acta Urologica Belgica. 1989;57(4):863-5.

144. Shishito S, Akazaki K, Sasano H, Irisawa S, Matsushita S. Rhabdomyosarcoma of the testis. Saishin-Igaku. 1965;20(7):1713-42.

145. Shipilov VI, Figurin KM. Rhabdomyosarcoma of the urethra and the penis. Urologiya i Nefrologiya. 1979(1):56-8.

146. M. L, P. T, B.A. C, C.C. G. Rhabdomyosarcoma of the urinary bladder and prostate in adults: A clinicopathologic study of 11 cases. Laboratory Investigation. 2009;89:178A.

147. Paner GP, McKenney JK, Epstein JI, Amin MB. Rhabdomyosarcoma of the urinary bladder in adults: predilection for alveolar morphology with anaplasia and significant morphologic overlap with small cell carcinoma. The American journal of surgical pathology. 2008;32:1022-8.

148. Al-Meshaan MK, Abdulhamed MN, Katchy KC. Rhabdomyosarcoma of the urinary bladder in an adult. Kuwait Medical Journal. 2008;40(1):70-1.

149. Rao MS, Radhakrishnan VV. Rhabdomyosarcoma of the urinary bladder in an adult. A case report. Indian Journal of Cancer. 1973;10(4):463-7.

150. Lauro S, Lalle M, Scucchi L, Vecchione A. Rhabdomyosarcoma of the urinary bladder in an elderly patient. Anticancer Research. 1995;15(2):627-9.

151. Krumerman MS, Katatikarn V. Rhabdomyosarcoma of the urinary bladder with intraepithelial spread in an adult. Archives of Pathology and Laboratory Medicine. 1976;100(7):395-7.

152. Zaslau S, Perlmutter AE, Farivar-Mohseni H, Chang WW, Kandzari SJ. Rhabdomyosarcoma of tunica vaginalis masquerading as hydrocele. Urology. 1001;65(5).

153. Phelan JT, Juardo J. Rhabdomyosarcomas. Surgery. 1962;52(4):585-91.

154. Soliman H, Ferrari A, Thomas D. Sarcoma in the Young Adult Population: An International View. Seminars in Oncology. 2009;36(3):227-36.

155. Herrera Puerto J, Dominguez Bravo C, Soler Fernandez JM, Laguna Alvarez E, Sevilla Zabaleta M, Caballero Gomez M, et al. [Sarcoma of the bladder. Report of 2 cases]. Actas urologicas espanolas. 1991;15:390-2.

156. Smith BH, Dehner LP. Sarcoma of the prostate gland. American Journal of Clinical Pathology. 1972;58(1):43-50.

157. Janet NL, May AW, Akins RS. Sarcoma of the prostate: a single institutional review. American Journal of Clinical Oncology. 2009;32(1):27-9.

158. Duhart JE, Padorno EA, Fredotovich NM. Sarcoma of the spermatic cord. Revista argentina de urologia y nefrologia. 1970;39(7):160-3.

159. Tannenbaum M. Sarcomas of the prostate gland. Urology. 1975;5(6):810-4.

160. Denis B, Duval F, Auvert J. Sarcomas of the spermatic cord. Journal d'Urologie et de Nephrologie. 1978;84(10-11):818-26.

161. Raney B, Jr., Heyn R, Hays DM, Tefft M, Newton WA, Jr., Wharam M, et al. Sequelae of treatment in 109 patients followed for 5 to 15 years after diagnosis of sarcoma of the bladder and prostate. A report from the Intergroup Rhabdomyosarcoma Study Committee. Cancer. 2387;71(7):2387-94.

162. Kazanowska B, Mikolajewska A, Balcerska A, Balwierz W, Bodalski J, Dluzniewska A, et al. [Soft tissue sarcoma of the bladder and prostate. A report of the Polish Paediatric Solid Tumour Group (PPSTG)]. [Polish]. Medycyna Wieku Rozwojowego. 1091;8(4 Pt 2):1091-8.

163. Zamolo G, Coklo M, Stifter S, Bosnar A, Markic D, Pavlovic-Ruzic I. Solid variant of alveolar rhabdomyosarcoma of the spermatic cord. Wiener Klinische Wochenschrift. 2005;117(9-10):323.

164. Rodriguez Garcia N, Llanes Gonzalez L, Pascual Mateo C, Berenguer Sanchez A. [Spermatic cord rhabdomyosarcoma in an adult]. [Spanish]. Archivos Espanoles de Urologia. 2005;58(9):956-9.

165. Beall ME, Young IS. Spermatic cord rhabdomyosarcoma: case report. Journal of Urology. 1977;117(6):807.

166. Merimsky O, Terrier P, Bonvalot S, Le Pechoux C, Delord JP, Le Cesne A. Spermatic cord sarcoma in adults. Acta Oncologica. 1999;38(5):635-8.

167. Latz S, Ellinger J, Goltz D, Marx C, Leuschner I, Muller SC, et al. Spindle cell rhabdomyosarcoma of the prostate. International Journal of Urology. 2013;20(9):935-7.

168. Grüschow K, Kyank U, Stuhldreier G, Fietkau R. Surgical repositioning of the contralateral testicle before irradiation of a paratesticular rhabdomyosarcoma for preservation of hormone production. Pediatric Hematology and Oncology. 2007;24(5):371-7.

169. Ng CS, Leung WT, Shui W, Cheng PN, Feng CS. Test and teach. Number Sixty. Diagnosis: Rhabdomyosarcoma presenting in the bone marrow. Pathology. 1989;21(2):88-9, 150.

170. Opot EN, Magoha GA. Testicular cancer at Kenyatta National Hospital, Nairobi. East African Medical Journal. 2000;77(2):80-5.

171. M. H, P. T, J. T, P. M, J. K. Testicular rhabdomyosarcoma weighing three kilograms. Prakticky Lekar. 1998;78:119-21.

172. Maldonado J, De Maldonado EF. Testicular rhabdomyosarcoma. Presentation of a case. Revista mexicana de urologia. 1965;24(4):401-8.

173. Stewart LH, Lioe TF, Johnston SR. Thirty-year review of intrascrotal rhabdomyosarcoma. British Journal of Urology. 1991;68(4):418-20.

174. Uekado Y, Ogawa T, Hirano A. [Treatment of five cases of bladder sarcoma]. Hinyokika kiyo Acta urologica Japonica. 1984;30:1085-93.

175. Stea B, Kinsella TJ, Triche TJ, Horvath K, Glatstein E, Miser JS. Treatment of pelvic sarcomas in adolescents and young adults with intensive combined modality therapy. International journal of radiation oncology, biology, physics. 1987;13:1797-805.

176. Kulkarni SV, Chaphekar PM. Tumors of the urinary bladder. Indian Journal of Cancer. 1974;11(2):151-61.

177. Dodge OG, Owor R, Templeton AC. Tumours of the male genitalia. Recent Results in Cancer Research. 1973;41:132-44.

178. Lioe TF, Biggart JD. Tumours of the spermatic cord and paratesticular tissue. A clinicopathological study. British Journal of Urology. 1993;71(5):600-6.

179. Wang X, Liu L, Tang H, Rao Z, Zhan W, Li X, et al. Twenty-five cases of adult prostate sarcoma treated at a high-volume institution from 1989 to 2009. Urology. 2013;82(1):160-5.

180. Silvestris N, Zito FA, Resta L, Popescu O, Rossi R, Montemurro S, et al. Unique Case of Giant Adult Paratesticular Spindle Cell Rhabdomyosarcoma. Urology. 2009;73(3):500-2.

181. Shanggar K, Muhilan P, Dublin N, George Lee EG, Azad Hassan AR. An unusual presentation of a rare Rhabdomyosarcoma of urinary bladder and prostate - case report. Journal of Health and Translational Medicine. 2007;10(2):57-9.

182. Townsend 3rd MF, Gal AA, Thoms WW, Newman JL, Eble JN, Graham Jr SD. Ureteral rhabdomyosarcoma. Urology. 1999;54(3):561.

183. Russo P. Urologic sarcoma in adults. Memorial Sloan-Kettering Cancer Center experience based on a prospective database between 1982 and 1989. Urologic Clinics of North America. 1991;18(3):581-8.

184. Ghyoot Y, Kaeckenbeeck B, Willem C. Vesical rhabdomyosarcoma of the adult. Acta Urologica Belgica. 1978;46(1):17-22.

Retroperitoneal references

1. Aassab R, Kharmoume S, Mahfoud T, Khmamouche MR, M'Rabti H, Errihani H. Primary embryonal botryoid-type rhabdomyosarcoma of the liver in adult: Case report and review of the literature. Journal Africain du Cancer. 2012;4(2):124-6.

2. Attili VSS, Dadhich HK, RamaRao C, Bapsy PP, Ramachandra C, Anupama G, et al. A case of primary rhabdomyosarcoma of the breast. Indian Journal of Surgery. 2007;69(4):201-2.

3. Batoroev YK, Nguyen G-K. Esophageal rhabdomyosarcoma: report of a case diagnosed by imprint cytology. Acta cytologica. 2006;50:213-6.

4. Becker V, Gaa J, Ott K, Seemann M, Becker K, Schmid R. A rare case of primary rectal rhabdomyosarcoma in an adult. Zeitschrift fur Gastroenterologie. 2006;44:1149-52.

5. Cobanoglu B, Kandi B, Okur I. Primary cutaneous rhabdomyosarcoma in an adult. Dermatologic Surgery. 2009;35(10):1573-5.

6. Colleoni M, Nelli P, Sgarbossa G, Pancheri F, Manente P. Primary cutaneous rhabdomyosarcoma in adults - Description of an uncommon aggressive disease. Acta Oncologica. 1996;35(4):494-5.

7. Fernando Val-Bernal J, Fernandez N, Gomez-Roman JJ. Spindle cell rhabdomyosarcoma in adults. A case report and literature review. [Review] [22 refs]. Pathology, Research & Practice. 2000;196(1):67-72.

8. Fox KR, Moussa SM, Mitre RJ, Zidar BL, Raves JJ. Clinical and pathologic features of primary gastric rhabdomyosarcoma. Cancer. 1990;66(4):772-8.

9. Haider N, Nadim MS, Piracha MN. Primary embryonal rhabdomyosarcoma of the liver in a young male. Jcpsp, Journal of the College of Physicians & Surgeons Pakistan. 2013;23(10):750-1.

10. Hays DM, Donaldson SS, Shimada H, Crist WM, Newton W.A, Jr., Andrassy RJ, et al. Primary and metastatic rhabdomyosarcoma in the breast: Neoplasms of adolescent females, a report from the Intergroup Rhabdomyosarcoma Study. Medical and Pediatric Oncology. 1997;29(3):181-9.

11. Ji GY, Mao H. Primary pulmonary rhabdomyosarcoma in an adult: a case report and review of the literature. [Review]: Journal of Zhejiang University SCIENCE B. 14(9):859-65, 2013 Sep.; 2013.

12. Kaplan AM, Creager AJ, Livasy CA, Dent GA, Boggess JF. Intra-abdominal embryonal rhabdomyosarcoma in an adult. Gynecologic Oncology. 1999;74(2):282-5.

13. Kyriazis AP, Kyriazis AA. Primary rhabdomyosarcoma of the female breast: Report of a case and review of the literature. Archives of Pathology and Laboratory Medicine. 1998;122(8):747-9.

14. Liang W, Xu S. Adult pancreatic rhabdomyosarcoma: One rear case and review of the literature. European Journal of Radiology Extra. 2011;79(1):e21-e2.

15. Marnewick J, Hulme-Moir M. Embryonal rhabdomyosarcoma of the rectum: report of a case and possible treatment option. Colorectal Disease. 2010;12(7 Online).

16. Mikubo M, Ikeda S, Hoshino T, Yokota T, Fujii A, Mori M. Sclerosing Rhabdomyosarcoma of a Chest Wall in an Adult: A Case Report and Review of the Literature. Annals of thoracic and cardiovascular surgery : official journal of the Association of Thoracic and Cardiovascular Surgeons of Asia. 2013.

17. Moody AM, Norman AR, Tait D. Paediatric tumours in the adult population: the experience of the Royal Marsden Hospital 1974-1990. Medical & Pediatric Oncology. 1996;26(3):153-9.

18. Prestidge BR, Donaldson SS. Treatment results among adults with childhood tumors: a 20-year experience. International Journal of Radiation Oncology, Biology, Physics. 1989;17(3):507-14.

19. Ramstead C, Buffam F, White V. Caruncular alveolar rhabdomyosarcoma in a woman previously treated for breast cancer. Archives of Ophthalmology. 2012;130(8):1087-8.

20. S. G-G, C. R, R.M. K, V. F, J. B. Primary alveolar rhabdomyosarcoma of the breast in a 16-year-old female patient. Breast. 1998;7:286-8.

21. Sato A, Hashimoto M, Moriyama J, Hanaoka M, Momose K, Kinowaki K, et al. Rhabdomyosarcoma of the duodenum: report of a case. Surgery Today. 2014;44(2):378-82.

22. Seidal T, Kindblom LG, Angervall L. Rhabdomyosarcoma in middle-aged and elderly individuals. Apmis. 1989;97(3):236-48.

23. Suárez-Vilela D, Izquierdo-Garcia FM, Alonso-Orcajo N. Epithelioid and rhabdoid rhabdomyosarcoma in an adult patient: A diagnostic pitfall [2]. Virchows Archiv. 2004;445(3):323-5.

24. Sundaresan N, Rosen G, Fortner JG, Lane JM, Hilaris BS. Preoperative chemotherapy and surgical resection in the management of posterior paraspinal tumors. Report of three cases. Journal of Neurosurgery. 1983;58(3):446-50.

25. Tanaka T, Harada M, Hara M, Sakai T, Kikyo S. Three cases of rhabdomyosarcoma originated from relatively unusual sites. Acta Pathologica Japonica. 1970;20(1):103-10.

26. Uphouse WJ, Lee YTM, Ronquillo AP, Fleet K. Rapid remission of a large pleomorphic rhabdomyosarcoma with radiation and a novel schedulre of simultaneous high-dose cisplatin. Selective Cancer Therapeutics. 1989;5(4):205-6.

27. Watanabe Y, Yamaguchi A, Isogai M, Kaneoka Y, Suzuki M, Ando H, et al. Treatment strategies for perianal rhabdomyosarcoma: report of two cases. [Review] [21 refs]. Surgery Today. 2004;34(8):719-24.

Chest references

1. Llombart-Cussac A, Pivot X, Contesso G, Rhor-Alvarado A, Delord JP, Spielmann M, et al. Adjuvant chemotherapy for primary cardiac sarcomas: the IGR experience. British journal of cancer. 1998;78:1624-8.

2. Dirican A, Kucukzeybek Y, Erten C, Somali I, Can A, Bayoglu IV, et al. Cardiac rhabdomyosarcoma of the left atrium. Wspolczesna Onkologia. 2014;18(1):73-5.

3. Yilmaz M, Kehlibar T, Arslan IY, Yilmaz HY, Tarhan IA, Ozler A. A case of primary cardiac rhabdomyosarcoma with surgical removal and mitral valve repair. Heart Surgery Forum. 2013;16(3).

4. Toyo-oka T, Murata K, Matsuya S. A case of primary rhabdomyosarcoma, replacing the atrioventricular node. Japanese Heart Journal. 1974;15(5):532-41.

5. Saenz NC, Ghavimi F, Gerald W, Gollamudi S, LaQuaglia MP. Chest wall rhabdomyosarcoma. Cancer. 1997;80:1513-7.

6. Panasuk DB, Bauer TL, Davies AL, Schneider C, Flynn C. Common malignancies with uncommon sites of presentation: case 1. Anterior mediastinal rhabdomyosarcoma. Journal of Clinical Oncology. 4455;21(23):4455-6.

7. Ryan MB, McMurtrey MJ, Roth JA. Current management of chest-wall tumors. The Surgical clinics of North America. 1989;69:1061-80.

8. Fan HG, Meng J, Pan SW, Zheng Z, Hu SS. Diagnosis, operation, recurrence, metastasis, and death: a case of primary cardiac rhabdomyosarcoma. Journal of Cardiac Surgery. 2009;24(4):480-2.

9. Ma J, Sun JP, Chen M, Zhang L, Xu N, Wang J, et al. Left atrial rhabdomyosarcoma. Circulation. 2014;129(21):27.

10. Tiyapant A, Tantranont R. Primary cardiac embryonal rhabdomyosarcoma: the first officially case reported of Thailand. Journal of the Medical Association of Thailand. 1991;74(3):176-80.

11. Satoh M, Horimoto M, Sakurai K, Funayama N, Igarashi K, Yamashiro K. Primary cardiac rhabdomyosarcoma exhibiting transient and pronounced regression with chemotherapy. American Heart Journal. 1458;120(6 Pt 1):1458-60.

12. Sokullu O, Sanioglu S, Deniz H, Ayoglu U, Ozgen A, Bilgen F. Primary cardiac rhabdomyosarcoma of the right atrium: case report. Heart Surgery Forum. 2008;11(2).

13. Turner A, Batrick N. Primary cardiac sarcomas. A report of three cases and a review of the current literature. International journal of cardiology. 1993;40:115-9.

14. Van de Wal HJ, Fritschy WM, Skotnicki SH, Lacquet LK. Primary cardiac tumors. Acta Chirurgica Belgica. 1988;88(2):74-8.

15. Orcioni GF, Ravetti JL, Gaggero G, Bocca B, Bisceglia M. Primary embryonal spindle cell cardiac rhabdomyosarcoma: Case report. Pathology Research and Practice. 2010;206(5):325-30.

16. Vyas V, Al Awadi S, Nemec J, El Khodary A, Francis IM, Muralidharan KC, et al. Primary mediastinal pleomorphic rhabdomyosarcoma: a case report. Medical Principles & Practice. 2008;17(2):154-6.

17. M. M, H.-J. H. Primary pleomorphic rhabdomyosarcoma of the lung - A case report and review of the literature. Zeitschrift fur Herz-, Thorax- und Gefasschirurgie. 2001;15:97-102.

18. Si D, Zhang B, Zhang X, Zhang M, Ni L, Yang P. Primary pulmonary artery rhabdomyosarcoma. Acta Cardiologica. 2011;66(3):391-4.

19. Geping Q, Qingyu X, Bing L, Zhaoquan S. Primary pulmonary rhabdomyosarcoma in adult: A case report. Journal of Medical Colleges of PLA. 2009;24(6):370-2.

20. Gupta A, Sharma MC, Kochupillai V, Kichendasse G, Gupta A, Atri S, et al. Primary pulmonary rhabdomyosarcoma in adults: case report and review of literature. Clinical Lung Cancer. 2007;8(6):389-91.

21. Skopin II, Serov RA, Makushin AA, Sazonenkov MA. Primary rabdomyosarcoma of the right atrium. Interactive Cardiovascular and Thoracic Surgery. 2003;2(3):316-8.

22. Suman KC, Sharma P, Singh H, Bal C, Kumar R. Primary rhabdomyosarcoma of pulmonary artery: 18F-FDG PET/CT for detecting recurrence in a rare tumor. Clinical Nuclear Medicine. 2013;38(3).

23. Rea F, Loy M, Bonavina L, Vigo M, Salmaso R, Calabro F. Primary rhabdomyosarcoma of the diaphragm. Report of a case presenting with hemothorax. Thoracic & Cardiovascular Surgeon. 1992;40(4):201-3.

24. Medeiros CW, Kondo W, Baptista I, Jr., Vizzotto AO, Jr., Noronha L, Hakim Neto CA. Primary rhabdomyosarcoma of the diaphragm: case report and literature review. [Review] [27 refs]. Revista do Hospital das Clinicas. 2002;57(2):67-72.

25. Melis M, Rosen G, Hajdu CH, Pachter HL, Raccuia JS. Primary rhabdomyosarcoma of the diaphragm: case report and review of the literature. [Review]. Journal of Gastrointestinal Surgery. 2013;17(4):799-804.

26. Mahalu W, Coltart DJ, Braimbridge MV. Primary rhabdomyosarcoma of the right ventricle. East African Medical Journal. 1980;57(10):696-9.

27. Cameron EWJ. Primary sarcoma of the lung. Thorax. 1975;30(5):516-20.

28. McAllister Jr HA. Primary tumors of the heart and pericardium. Pathology Annual. 1979;14 Pt 2:325-55.

29. Yokoi Y, Miyaji K, Ochi Y, Munemasa M, Kato G, Nakai M, et al. Rapidly progressing multiple cardiac rhabdomyosarcoma. Journal of Echocardiography. 2010;8(2):62-4.

30. Midorikawa Y, Kubota K, Mori M, Koyama H, Aihara N, Makuuchi M, et al. Rhabdomyosarcoma of the diaphragm: report of an adult case. [Review] [12 refs]. Japanese Journal of Clinical Oncology. 1998;28(3):222-6.

31. Sochocky S. Rhabdomyosarcoma of the heart. Minnesota Medicine. 1971;54(9):747-50.

32. Vujin B, Benc D, Srdic S, Bikicki M, Vuckovic D, Dodic S. Rhabdomyosarcoma of the heart. Herz. 2006;31(8):798-800.

33. Margolis J, Arganaras E. Rhabdomyosarcoma of the heart (case report). Journal of the American Geriatrics Society. 1970;18(2):183-7.

34. Matloff JM, Bass H, Dalen JE. Rhabdomyosarcoma of the left atrium. Physiologic responses to surgical therapy. Journal of Thoracic & Cardiovascular Surgery. 1971;61(3):451-5.

35. Williams AO, Martinson FD, Alli AF. Rhabdomyosarcoma of the upper respiratory tract in Ibadan, Nigeria. British Journal of Cancer. 1968;22(1):12-8.

36. Suster S, Moran CA, Koss MN. Rhabdomyosarcomas of the anterior mediastinum: Report of four cases unassociated with germ cell, teratomatous, or thymic carcinomatous components. Human Pathology. 1994;25(4):349-56.

37. Qi Y, Chang B, Pang L, Liu C, Li F. Solid alveolar rhabdomyosarcoma with spindle-shaped cells and epithelial differentiation of the mediastinum in a 68-year-old man: A case report and literature review. Journal of Cancer Research and Therapeutics. 2011;7(3):353-6.

38. Kamiya H, Yasuda T, Nagamine H, Sakakibara N, Nishida S, Kawasuji M, et al. Surgical treatment of primary cardiac tumors: 28 years' experience in Kanazawa University Hospital. [Review] [26 refs]. Japanese Circulation Journal. 2001;65(4):315-9.

39. Grandmougin D, Fayad G, Decoene C, Pol A, Warembourg H. Total orthotopic heart transplantation for primary cardiac rhabdomyosarcoma: Factors influencing long-term survival. Annals of Thoracic Surgery. 2001;71(5):1438-41.

Extremities references

1. Cronin CT, Keel SB, Grabbe J, Schuler JG. Adult rhabdomyoma of the extremity: a case report and review of the literature. [Review] [28 refs]. Human Pathology. 1074;31(9):1074-80.

2. McClemont JMF, Webb JN. Tumours arising in skeletal muscle in adults. Journal of Pathology. 1976;118(2):113-20.

3. Oda Y, Tsuneyoshi M, Hashimoto H, Iwashita T, Ushijima M, Masuda S, et al. Primary rhabdomyosarcoma of the iliac bone in an adult: a case mimicking fibrosarcoma. [Review] [21 refs]. Virchows Archiv A, Pathological Anatomy & Histopathology. 1993;423(1):65-9.

4. Thomas F, Lipton JF, Barbera C, Vigorita VJ, Bryk E. Primary rhabdomyosarcoma of the humerus. A case report and review of the literature. Journal of Bone and Joint Surgery - Series A. 2002;84(5):813-7.
