# Supplementary material for: Management of Low and Intermediate Risk Adult Rhabdomyosarcoma: A Pooled Survival Analysis of 553 Patients
Source: Sci Rep. 2018 Jun 19;8:9337. doi: 10.1038/s41598-018-27556-1 (PMC6008292; doi:10.1038/s41598-018-27556-1)
Supplement: Supplementary file 3 — Supplementary S3 [file 41598_2018_27556_MOESM3_ESM.pdf]

Management of Low and Intermediate Risk Adult Rhabdomyosarcoma: A Pooled Survival Analysis of 553 Patients

Maha AT Elsebaie[1], Mohamed Amgad[†2], Ahmed Elkashash[†3], Ahmed Saber Elgebaly[4,5], Gehad Gamal El Ashal[3,5], Emad Shash[6], Zeinab Elsayed*[7]

1 Faculty of Medicine, Ain Shams University, Cairo, Egypt.

2 Department of Biomedical Informatics, Emory University School of Medicine, Atlanta, GA, USA.

3 Kasr Al Ainy School of Medicine, Cairo University, Cairo, Egypt

4 Faculty of Medicine, Al-Azhar University, Cairo, Egypt.

5 Medical Research Education and Practice Association (MREP)

6 Medical Oncology Department, National Cancer Institute, Cairo University, Cairo, Egypt.

7 Adult Sarcoma Division, Clinical Oncology Department, Ain Shams University Hospitals, Cairo, Egypt.

† Authors contributed equally

Definitions for the Data Extraction Form

1. Histological subtype: Tumor histology was classified into: Botryoides/Spindle cell RMS (favorable histology), Embryonal RMS (favorable histology), and Alveolar RMS, mixed embryonal/alveolar RMS, Pleomorphic RMS, Anaplastic RMS and Undifferentiated/NOS RMS (unfavorable histologies).[1]
2. Tumor size: Tumor size was assessed based on maximal tumor dimension.
3. Pre-treatment Tumor Stage:
Pretreatment tumor stage had to be reported as defined in the Intergroup Rhabdomyosarcoma Study Group (IRSG) [2] and/or the International Union Against Cancer (UICC) TNM staging guidelines.[3] For few patients, however, tumor stages were reported using the American Joint Committee on Cancer (AJCC) staging system for adult Soft Tissue Sarcoma.[4] Because the AJCC staging takes into consideration different parameters from the IRS and UICC systems (e.g. histologic grade), those patients (n=25) were excluded from the tumor stage analysis.
In few circumstances, we attempted to conclude the T-stage according to the IRSG staging guidelines. For example, if a study reported the following for a primary RMS of the larynx *"MRI of the neck revealed a hypervascular malignant mass showing extra-laryngeal and subglottic extension"* but without explicitly assigning the patient a T-stage, we therefore assign this patient a T2-stage (*tumor extend outside site of origin*). For such cases, an orange highlight was added in the patient database to mark any indirect conclusions, accompanied by quoting the original text and our rationale for such conclusion. Decisions on "*site of origin*" and "*local tumor extension*" were made using the standardized definitions for "site of origin" in the European Pediatric Soft-Tissue Sarcoma Study Group (EpSSG) guidelines and the AJCC cancer staging manual, 7th-edition. [5, 6].
T stage:
T1: Tumor confined to site of origin
T2: Tumor extended outside to site of origin
N stage:
N0: *Radiologically/Pathologically proven negative regional or distant LN involvement.
*Microscopic examination after LN sampling.
*Microscopic examination after LN dissection.
N1: Regional LN involvement (Distant LN involvement is considered distant metastasis M1)
M stage: All patients must be M0.
4. Post-surgical Clinical Group: The IRS Clinical Group classification (IRSG) system was used to determine the post-surgical tumor extent and margin status.[2]
How does the IRS grouping system work? The surgical-pathologic grouping system categorizes patients according to the extent of disease remaining after the initial surgical procedure(s) but before beginning chemotherapy and radiation therapy. [7] E.g.
1- If the patient's initial therapy was chemotherapy/chemo-radiotherapy → then the initial surgical procedure done was biopsy only → the patient is assigned group III.
2- If the patient's initial therapy was surgery → then the patient is assigned a group after his primary operation or primary reoperation (with conditions mentioned below).
Types of Surgery?
*Primary operation: First surgical procedure done to achieve complete resection.
*Primary reoperation: Surgical procedure done to achieve complete resection of the residual disease after the primary operation (incomplete resection) and before other therapies (Radiotherapy or Chemotherapy).
*Secondary operation: Surgical procedure done to achieve complete resection of a residual disease after neo-adjuvant chemotherapy.

When to assign a patient a post-surgical group?

Patients are assigned to a group after the primary operation or the primary re-operation, provided that the primary reoperation was done within 42 days of the primary operation (incomplete resection) and before the start of any chemotherapy [8, 9]. The primary reoperation should, therefore, be considered the definitive operation and patients should be assigned to a group after this operation.

Post-surgical clinical groups:

<table>
<tr><th>Group</th><th>Definition</th><th>Other Names</th><th colspan="2">subgroups</th></tr>
<tr><td rowspan="2">Group I</td><td rowspan="2">*N0 M0,
*Microscopic complete resection,
*Microscopic negative surgical margins</td><td rowspan="2">Radical resection
R0</td><td>IA</td><td>Localized to organ of origin: T1</td></tr>
<tr><td>IB</td><td>Infiltration outside muscle or organ of origin: T2</td></tr>
<tr><td rowspan="3">Group II</td><td rowspan="3">*Macroscopic complete resection.
*Evidence of regional disease</td><td rowspan="3">Marginal resection
R1</td><td>IIA</td><td>N0,
Microscopic positive surgical margins</td></tr>
<tr><td>IIB</td><td>N1,
Microscopic negative surgical margins</td></tr>
<tr><td>IIC</td><td>N1,
Microscopic positive surgical margins</td></tr>
<tr><td rowspan="2">Group III</td><td rowspan="2">Incomplete resection</td><td rowspan="2">Intralesional resection
R2</td><td>IIIA</td><td>Biopsy only</td></tr>
<tr><td>IIIB</td><td>N1 or Gross residual disease</td></tr>
<tr><td>Group IV</td><td>Distant metastasis from the start</td><td></td><td colspan="2"></td></tr>
</table>

5. Extent of Local surgery: Based on definitions of surgery and surgical guidelines in European Pediatric Soft-Tissue Sarcoma Study Group (EpSSG) RMS protocols.[5] Examples are the following:

**Surgery category**	**Radical/ Mutilating surgery**	**Gross resection**	**Incomplete resection**
**Tumor site**			
**For ANY SITE**		1-Tumorectomy/ local tumor excision.<br>2-Excisional biopsy.<br>3-Gross total resection	1-Biopsy Only<br>2-Debulking surgery
**1-Head and Neck**	1- Major resection of the face e.g:<br>Total mandibulectomy<br>Composite resection (commando operation)<br>2- Total Laryngectomy	1-Partial-laryngectomy.<br>2-Hemi-mandibulectomy	
**2-Orbit**	Orbital exenteration		Enucleation

**3-Genitourinary: Bladder and prostate**	1-Total cystectomy<br>2-Total prostatectomy<br>3-Pelvic exenteration with definitive intestinal or urinary diversion	1-Partial prostatectomy<br>2-Partial cystectomy	1-Biopsy Only<br>2-Debulking surgery
**4-Genitourinary: Para-testicular**	1-Pelvic exenteration with definitive intestinal or urinary diversion<br>2-Hemi-pelvectomy	1-Orchidectomy +/- hemiscrotectomy<br>2-Radical orchiectomy	
**5-Chest wall**	Pneumonectomy	1-Tumorectomy/ local tumor excision.<br>2-Excisional biopsy.	
**6-Extremities**	R0 resections<br>1-Amputation<br>2-Compartmental resection: En-bloc resection of the tumor with the entire muscular or anatomical compartment.<br>3-Wide en-bloc resection: the removal of the tumor with its pseudo-capsule and a margin of normal tissue.<br>4-Major muscular resection leading to important functional impairment	1-R0 or R1 resections<br>(limb preserving surgery)<br>2- Anatomical wide resection/ Gross total resection.	
**7-Abdomen/ Pelvis**	Entire compartmental resection		
**8-Gynecologic: Vagina/ CX/ Uterus/adenexae**	1-Hysterectomy with bilateral salpingo-oophorectomy with or without pelvic/para-aortic lymph node dissection.<br>2-Pelvic exenteration with or without definitive intestinal or urinary diversion.	1-Wide Local excision.<br>2-Polypectomy,<br>3-Conization<br>4-LEEP procedure<br>5-Polypectomy, Endometrial and endocervical curettages<br>6-Simple Trachelectomy (removal of the cervix, not the parametria)<br>7-Radical Trachelectomy (radical removal of the uterine cervix, adjacent parametria, 1-2 cm vaginal cuff with 1 cm free margin and preservation of corpus uteri.<br>8-Vaginectomy<br>9-Hemivulvectomy	

6. Response to Chemotherapy: Response to chemotherapy was assessed according to RECIST guidelines namely, change in the primary tumor size on pre/post therapy CT and MRI scans and/or presence of residual disease at time of resection.[10]

To Whom Tumor response could be assessed?

1- Primary chemotherapy/chemo-radiotherapy (No surgery done).
2- Neoadjuvant Chemotherapy.
2- Adjuvant Chemotherapy for patients with Group III disease (Incomplete Excision).

To Whom Tumor response couldn't be assessed? (not applicable)

1- Complete resection of localized disease before initiation of therapy (Group I)
2- Refusal to complete course of chemotherapy
3- Chemotherapy related deaths.

**Response**	**Explanation**
Complete response (CR)	Complete disappearance of all visible disease.
Partial response (PR)	Tumor volume reduction >66% but <100%
Minimal response (MR)	Tumor volume reduction >33% but <66%
Stable disease (SD)	No criteria for PR or PD (<33% tumor volume reduction)
Disease Progression (PD)	Any increase >20% in volume<br>Appearance of one or more new lesions

7. Color Codes

**RED**	Query case, Need to contact the author(s) for further explanations.
**BLUE**	A Case report with important observations made on it, such as very long survival or remarkable family history.
**ORANGE**	Our rationale for any indirect conclusions made from data reported in the original article accompanied with quoting the original text.
**YELLOW**	Email sent for missing information and/or updated follow up data.
**GREEN**	Missing items from the original article.

8. References

1. Newton WA, Jr., Gehan EA, Webber BL, Marsden HB, van Unnik AJ, Hamoudi AB, et al. Classification of rhabdomyosarcomas and related sarcomas. Pathologic aspects and proposal for a new classification--an Intergroup Rhabdomyosarcoma Study. Cancer. 1995;76(6):1073-85.
2. Lawrence W, Jr., Anderson JR, Gehan EA, Maurer H. Pretreatment TNM staging of childhood rhabdomyosarcoma: a report of the Intergroup Rhabdomyosarcoma Study Group. Children's Cancer Study Group. Pediatric Oncology Group. Cancer. 1997;80(6):1165-70.
3. Pedrick TJ, Donaldson SS, Cox RS. Rhabdomyosarcoma: the Stanford experience using a TNM staging system. J Clin Oncol. 1986;4(3):370-8.
4. National Comprehensive Cancer Network. NCCN Clinical Practice Guidelines in Oncology; Soft Tissue Sarcoma Version 2.2017 2017 [cited 2017 Aug 2]. Available from: https://www.nccn.org/professionals/physician_gls/pdf/sarcoma.pdf.
5. Bisogno G, Bergeron C, Jenney M. European paediatric Soft Tissue Sarcoma Study Group RMS 2005–a protocol for non-metastatic rhabdomyosarcoma, 2005. 2005.
6. Edge SB, Compton CC. The American Joint Committee on Cancer: the 7th edition of the AJCC cancer staging manual and the future of TNM. Ann Surg Oncol. 2010;17(6):1471-4.
7. Raney RB, Maurer HM, Anderson JR, Andrassy RJ, Donaldson SS, Qualman SJ, et al. The Intergroup Rhabdomyosarcoma Study Group (IRSG): Major Lessons From the IRS-I Through IRS-IV Studies as Background for the Current IRS-V Treatment Protocols. Sarcoma. 2001;5(1):9-15.
8. Crist WM, Anderson JR, Meza JL, Fryer C, Raney RB, Ruymann FB, et al. Intergroup rhabdomyosarcoma study-IV: results for patients with nonmetastatic disease. J Clin Oncol. 2001;19(12):3091-102.
9. Maurer HM, Gehan EA, Beltangady M, Crist W, Dickman PS, Donaldson SS, et al. The Intergroup Rhabdomyosarcoma Study-II. Cancer. 1993;71(5):1904-22.
10. Eisenhauer EA, Therasse P, Bogaerts J, Schwartz LH, Sargent D, Ford R, et al. New response evaluation criteria in solid tumours: revised RECIST guideline (version 1.1). Eur J Cancer. 2009;45(2):228-47.
