# Supplementary material for: Management of Low and Intermediate Risk Adult Rhabdomyosarcoma: A Pooled Survival Analysis of 553 Patients
Source: Sci Rep. 2018 Jun 19;8:9337. doi: 10.1038/s41598-018-27556-1 (PMC6008292; doi:10.1038/s41598-018-27556-1)
Supplement: Supplementary file 4 — Supplementary S4 [file 41598_2018_27556_MOESM4_ESM.pdf]

# Management of Low and Intermediate Risk Adult Rhabdomyosarcoma: A Pooled Survival Analysis of 553 Patients


Maha AT Elsebaie[1], Mohamed Amgad[†2], Ahmed Elkashash[†3], Ahmed Saber Elgebaly[4,5], Gehad Gamal El Ashal[3,5], Emad Shash[6], Zeinab Elsayed*[7]

1 Faculty of Medicine, Ain Shams University, Cairo, Egypt.

2 Department of Biomedical Informatics, Emory University School of Medicine, Atlanta, GA, USA.

3 Kasr Al Ainy School of Medicine, Cairo University, Cairo, Egypt

4 Faculty of Medicine, Al-Azhar University, Cairo, Egypt.

5 Medical Research Education and Practice Association (MREP)

6 Medical Oncology Department, National Cancer Institute, Cairo University, Cairo, Egypt.

7 Adult Sarcoma Division, Clinical Oncology Department, Ain Shams University Hospitals, Cairo, Egypt.

*† Authors contributed equally*

## Supplementary 4.1: Categorization System for Different Chemotherapy Categories

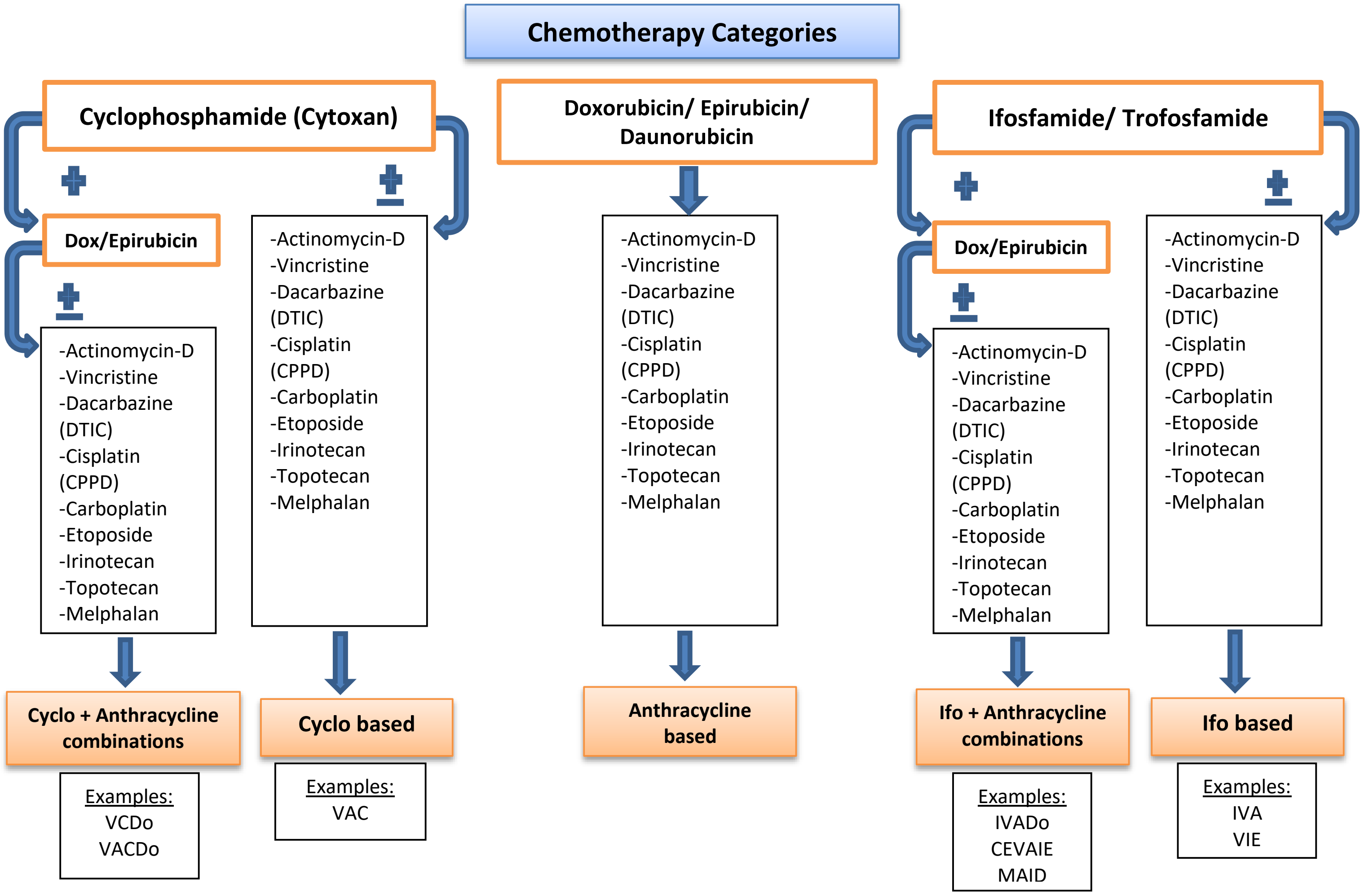


| | |
|---|---|
| **Others** | No Dox or IFo or Cyclo<br>Example: VA (Vincristine/ Actinomycin-D) |
| **Cyclo+Dox+Ifo Combination** | A combination containing all three drugs |

VAC: Vincristine, Actinomycin-D, Cyclophosphamide.
VACDo: VAC + Doxorubicin
IVA: Ifosfamide, Vincristine, Actinomycin-D.
IVADo: IVA + Doxorubicin
VIE: Vincristine, Ifosfamide, Etoposide.
CEVAIE: carboplaifotin, etoposide, vincristine, Actinomycin-D, ifosfamide, epirubicin.
MAID: mesna, doxorubicin, ifosfamide, dacarbazine.

## Supplementary 4.2: Quality assessment Criteria

| Criteria for Assessment | High quality (pool data for survival-analysis) | Intermediate quality (Email principal author for missing data) | Low quality (excluded from the analysis) |
|---|---|---|---|
| **Patient population clearly identified** (*Adult > 16 y.o; primary, pathologically proven, non-metastatic RMS*) | Yes | Yes | Any (e.g. Excluded studies are those describing rhabdomyosarcoma as a teratomatous tumor or mixed tumor) |
| **Minimal demographic and clinicopathological characteristics** are clearly specified for individual patients? *Age, sex, subtype, stage, staging system, primary site*. | Yes | yes | Any |
| **Treatment modality** mentioned? | Yes | Yes | No |
| **Types of chemotherapy** mentioned? | Yes | yes | No |
| **Types of chemotherapy** specified for individual patients rather than for groups? | Yes | Any (Only if they mention that exact chemotherapy regimen were available for all patients | No |
| **Timing of chemotherapy** mentioned? | Yes | Any | No |
| **Intervention data** specified for individual patients rather than for groups? | Yes | Any | No |
| **Follow up** data available for individual patients? | Yes | Yes | No (e.g. recent cases) |
| **Missing data of interest (other than the aforementioned data)** *(e.g. tumor size, T status, Nodal status, timing and dose of radiotherapy)* | No | Any | Any |
| **Objective?** Did the study aim to compare the efficacy of different chemotherapy regimens/ treatment protocols/ treatment modalities on survival outcomes? | yes | yes | No (e.g. Clinicopathological, ultra-structural, immunophenotypic and genetic studies) |

**Supplementary 4.3: Distribution of the different age groups between low and intermediate risk patients**

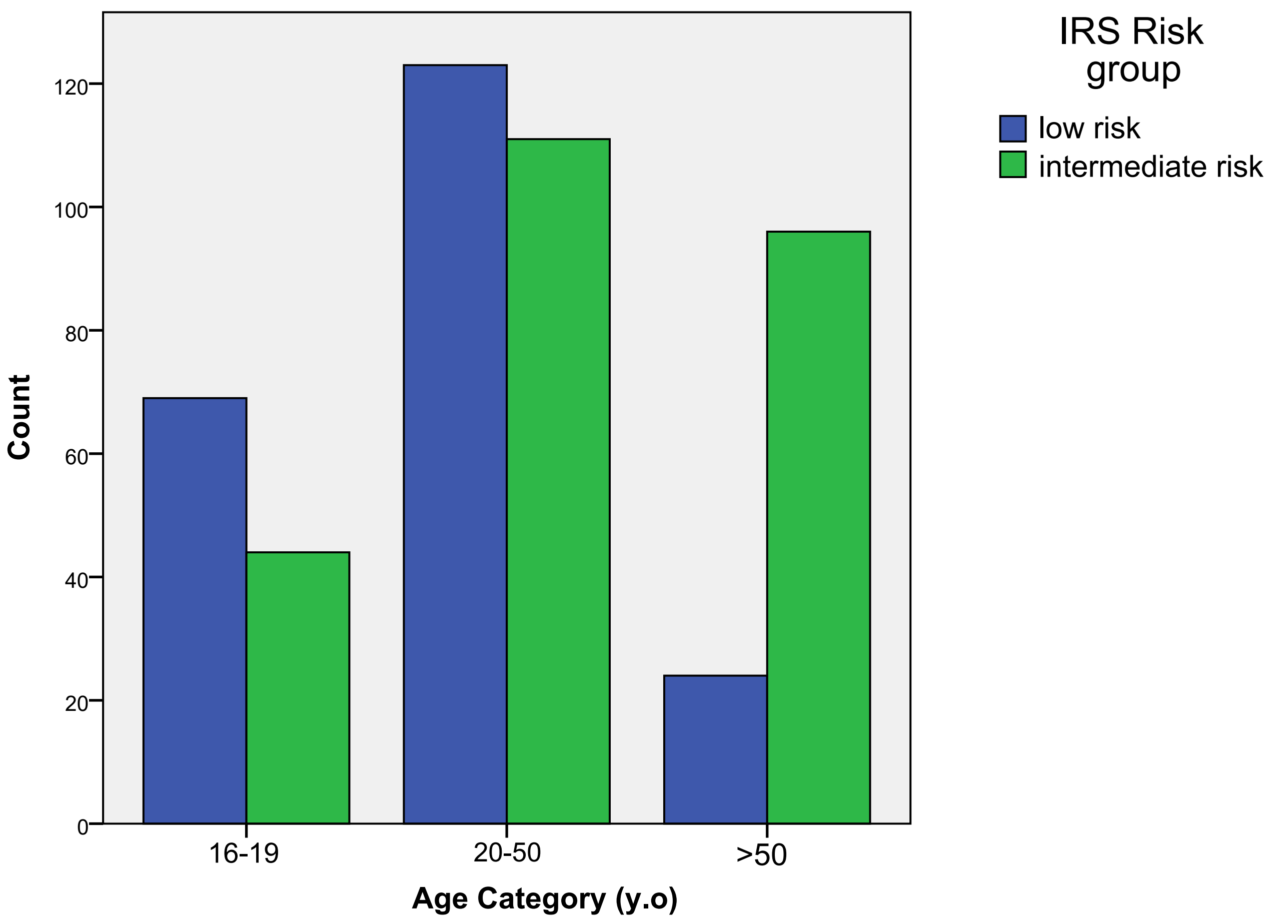

**Supplementary 4.4: Pairwise comparisons of the effect different Chemotherapeutic regimens on PFS and OS**

| **Chemotherapy Category** | **N.** | **5-y PFS** | Cyclo based | Cyclo and Anthracycline based | Ifo based | Ifo and Anthracycline based | Anthracycline based | Cyclo and Ifo and Anthracyclin combination$^{\pi}$ | Other/ Unknown |
|---|---|---|---|---|---|---|---|---|---|
| | | | | ***p*-value of Pairwise Comparisons (Gehan-Wilcoxon statistic)** | | | | | |
| Cyclo based | 85 | 64% | | 0.198 | 0.645 | 0.826 | 0.091 | 0.227 | 0.05 |
| Cyclo and Anthracycline based | 78 | 74% | 0.198 | | 0.671 | 0.194 | 0.016 | 0.594 | 0.001 |
| Ifo based | 15 | * | 0.645 | 0.671 | | 0.54 | 0.08 | 0.324 | 0.163 |
| Ifo and Anthracycline based | 52 | 63% | 0.826 | 0.194 | 0.54 | | 0.135 | 0.205 | 0.165 |
| **Anthracycline based** | 18 | 47% | **0.091** | **0.016** | 0.08 | 0.135 | | **0.037** | 0.554 |
| Cyclo and Ifo and Anthracycline combination | 14 | 80% | 0.227 | 0.594 | 0.324 | 0.205 | 0.037 | | 0.038 |
| Other/Unknown | 115 | 45% | 0.05 | 0.001 | 0.163 | 0.165 | 0.554 | 0.038 | |

PFS: Progression-free survival; * No 5y-PFS available (maximum PFS months available for this group is 50 months).
$^{\pi}$ 14 patients received intensified chemotherapy consisting of cyclophosphamide/anthracycline/ifosfamide combination-based regimen, with excellent 5y-PFS of 80% (*p*=0.037)
Cyclo: Cyclophosphamide; Ifo: Ifosfamide; Anthracycline: Doxorubicin/ Epirubicin/ Daunorubicin

| **Chemotherapy Category** | **N.** | **5-y OS** | Cyclo based | Cyclo and Anthracyclin e based | Ifo based | Ifo and Anthracycline based | Anthracycline based | Cyclo and Ifo and Anthracycline combination | Other/Un known |
|---|---|---|---|---|---|---|---|---|---|
| | | | | ***p*-value of Pairwise Comparisons (Gehan-Wilcoxon statistic)** | | | | | |
| Cyclo based | 85 | 56% | | 0.014 | 0.689 | 0.349 | 0.022 | 0.363 | 0.159 |
| Cyclo and Anthracycline based | 78 | 78% | **0.014** | | 0.44 | **0.003** | 0.000 | 0.659 | **0.000** |
| Ifo based | 15 | 71% | 0.689 | 0.44 | | 0.381 | 0.087 | 0.563 | 0.295 |
| Ifo and Anthracycline based | 52 | 57% | 0.349 | 0.003 | 0.381 | | 0.232 | 0.217 | 0.847 |
| **Anthracycline based** | 18 | 36% | **0.022** | **0.000** | 0.087 | 0.232 | | **0.039** | 0.151 |
| Cyclo and Ifo and Anthracycline combination | 14 | 48% | 0.363 | 0.659 | 0.563 | 0.217 | 0.039 | | 0.12 |
| Other/Unknown | 115 | 42% | 0.159 | 0.000 | 0.295 | 0.847 | 0.151 | 0.12 | |

**Supplementary 4.5: Distribution of the chemotherapy response between low and intermediate risk patients**

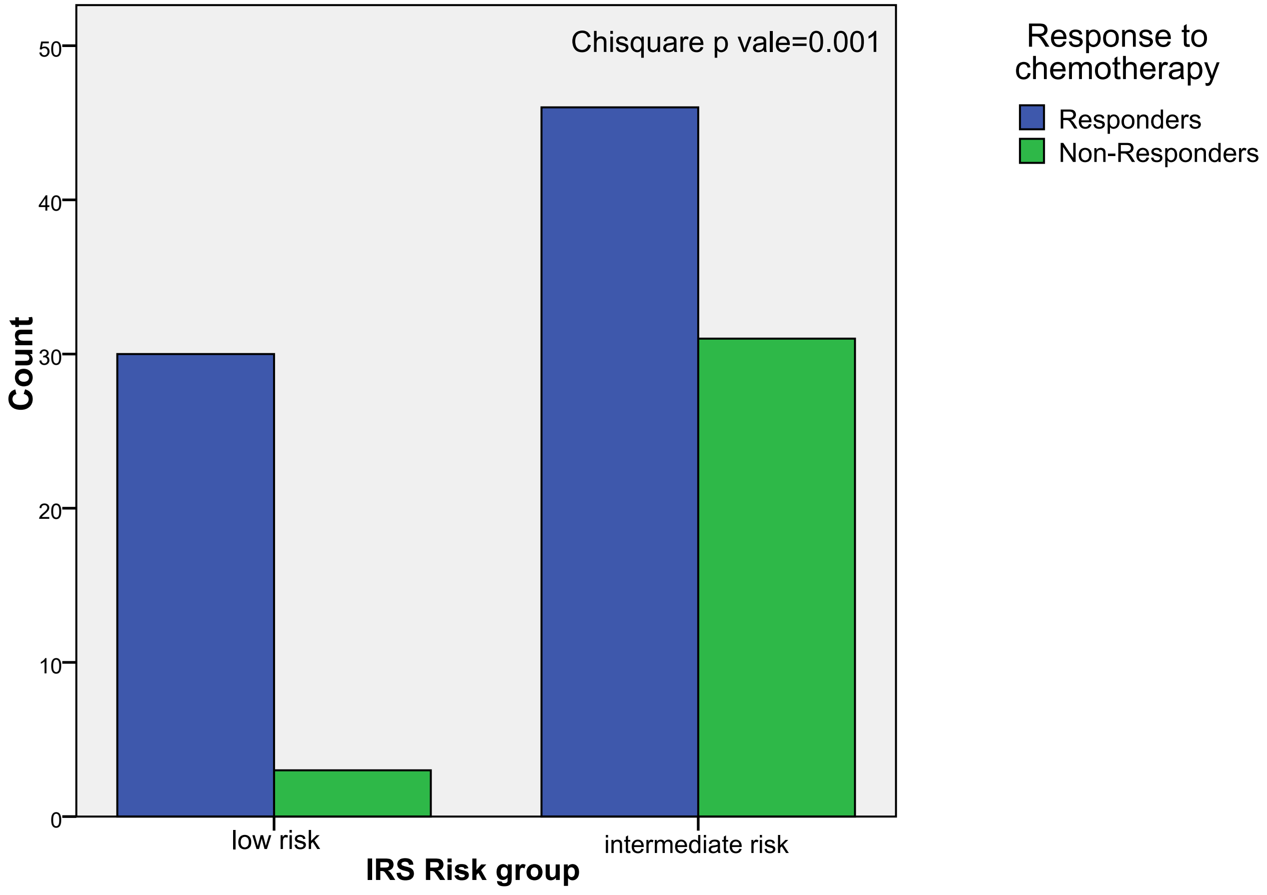

**Supplementary 4.6: OS outcomes of intermediate risk patients who showed response (CR/PR) vs. no response/disease progression (NR/PD)**

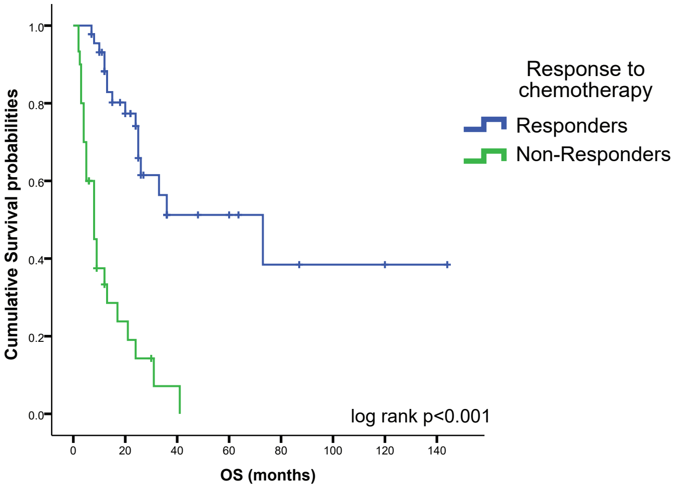


**Supplementary 4.7: Local control of PR/NR cohort treated with delayed complete resection vs. definitive CRT**

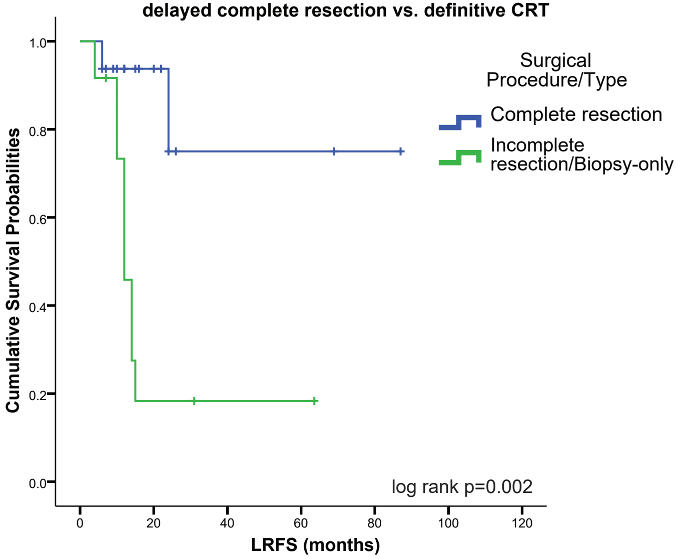

## Supplementary 4.8: Comprehensive Comparison of the largest retrospective analysis Performed on adult RMS patients as of September, 2016

| Study | La Quaglia et al, 1994 | Esnaola et al, 2001 | Hawkins et al, 2001 | Little et al, 2002 | Ferrari et al, 2003 | Simon et al, 2003 | Sultan et al, 2009 | Gerber et al, 2013 | Van Gaal et al, 2012 | Dumont et al, 2013 | Kojima etal, 2012 |
|---|---|---|---|---|---|---|---|---|---|---|---|
| **Patient Source** | Memorial Sloan-Kettering Cancer Center | Dana Farber Cancer Institute | Memorial Sloan-Kettering Cancer Center | M.D. Anderson Cancer Center | Milan, Italy | USA | SEER Database | Memorial Sloan-Kettering Cancer Center | Netherlands | MD Anderson Cancer Center | Japan |
| **Population size** | 290 | 39 | 84 | 82 | 171 | 39 | 1071 | 138 | 169 | 239 | 36 |
| **Population type** | Mixed (Target is 133) | Target patients analyzed separately | Mixed (47M0, 37M1) analysis | Target | Target patients analyzed separately | Mixed (33M0, 6M1) analysis | Target patients analyzed separately | Target patients analyzed separately | Only Emb and Alv | Target patients analyzed separately | Target patients analyzed separately |
| **Non-metastatic adults** | 133 | 26 | 47 | 82 | 149 | 33 | 617 | 94 | 34 (51 adults, 17 metastatic) | 163 | 22 |
| **5y-OS for non-metastatic patients** | | 44% | N/A (Median DSS survival is 35 vs. 14) | 44% | 45.7% | N/A (35% for whole cohort) | 47% | 45% | 40±13% (E, non-metastatic) | 44.1% with 95% CI 0.362–0.516 | 55-60%* *Deducted from KM curve |
| **Received Chemo** | | 24 | - | 56 | 110 | 22 | N/A | 81 | | 146 | 22 |
| **Type of Chemo** | | 14 patients received **VAC** while 10 patients received doxorubicin-based or ifosfamide-containing regimens. | | Vin and Cyclo with Act **(VAC)** or DOX **(VACA).** | **74 received a chemotherapy score of 1** → multidrug regimen consisting of cyclophosphamide or ifosfamide, with doxorubicin, epirubicin, and/or dactinomycin, with without vincristine (and with or without other drugs, such as dacarbazine, cisplatin, carboplatin, and etoposide); and lasted for 8 cycles or more. **They assigned scores of 0.4, 0.5, and 0.6** to chemotherapy regimens lasting 2, 4, | The most commonly employed agents were VAC in seven, actinomycin-D alone in four and VA in three patients. | | OS rates were significantly better for adults patients treated according to a prospective RMS protocol compared to patients treated off protocol. In comparing the two patient groups, they found that protocol patients were more likely to receive cyclophosphamide, doxorubicin, and vincristine compared to non-protocol patients | | **39% received Doxorubicin-based (No ifo/cyclo),** 3% had Ifo-based, 12% had Ifo/Dox based regimens, 20% Any Act-D (No Dox, Ifo), 11% Unknown. "Any actinomycin D, No doxorubicin/ifosfamide" were significantly associated with longer RFS but lost significance on multivariate level. | VAC or VAC-like chemotherapy. according to IRS-IV (D9803) protocols. The VAC-like regimen includes the following regimens: vincristine, d-actinomycin, and either ifosfamide, etoposide, or doxorubicin. |

| | | | | | | | | | | | |
|---|---|---|---|---|---|---|---|---|---|---|---|
| | | | | | or 6 cycles, and a score of 0.5 to chemotherapy regimens that did not include cyclophosphamide or ifosfamide (i.e., doxorubicin-based). | | | (71% vs 20%, p<0.0001) | | | |
| **5-y OS for non-metastatic patients who received chemotherapy** | | 5-y OS rate 44% | | 10-y OS rate 47% | 5-y OS 49.9%: **Score 1** 5y-OS 11.5% for **score 0.4-0.5** Five-year OS 45.7% for entire cohort of non-metastatic patients. | | | 3-y, 4-y and 5-y OS (71%, 61%, and 54%, respectively). for non-metastatic patients treated on protocol. On MVA, risk group (intermediate vs low, HR 2.78, 95% CI 1.40-5.53, PZ.004) and protocol participation were independent prognosticators. | 5-year OS 49.2±15.4% | Median OS of 3.8 years (95% CI 2.8–7.6). The 2-year OS was 0.660 with 95% CI 0.582–0.727, and the 5-year OS was 0.441 with 95% CI 0.362–0.516. | |
| **5-yPFS/ EFS/DMFS for patients who received chemotherapy** | | | | 10y-DMFS 59% | 5y-DMFS 64.1% **Score 1** 5y-DMFS 27.8% for **score 0.4-0.5** Five-year DMFS 56.2% for entire cohort of non-metastatic patients. Five-year EFS 32.9% for entire cohort of non-metastatic patients. | | | DM rate at 5 years was 42%. The failure rates at 5 years for patients with nonmetastatic disease were 34% for local failure and 42% for distant failure. | | The median RFS was 1.9 years (95% CI 1.3–2.8 years). the 5-year RFS rate was 0.362 (95% CI 0.288–0.436). | Median PFS of localized children and adults was as follows: 166.9 versus 22.4 months (p = 0.005). and metastatic disease, 13.3 versus 13.3 months (p = 0.949), |
| **Age (y)** | | | | | | | | | | | |
| **Univariate** | Significant OS | Not significant OS (<26y; >26y) | Significant: Age (<20, >20) on DSS, DMFS and LRFS. | Not significant (<28; >28) on OS, DFS, LRFS, DMFS | Not significant (19-30; 31-60; >60) on OS, FFS (p=0.9393) | Not significant | Significant on OS, DSS | Significant (continuous variable) on OS (p=0.02) | Significant (<16, >16) on DSS, PFS | Significant (<20, 20-50, >50) OS, RFS | Significant (<21 vs. >21) on PFS |

| | | | | | | | | | | | |
|---|---|---|---|---|---|---|---|---|---|---|---|
| **Multivariate** | Significant OS (p=0.0001) | Not significant OS | Significant: Age (<20, >20) on DSS, DMFS and LRFS. | Not significant on OS, DFS, LRFS, DMFS | Not tested | Not significant | Significant on OS, DSS | Not significant OS | The only significant factor for PFS. Significant on DSS | Significant OS, RFS | Significant on PFS. Not significant on OS |
| **Primary Tumor site (Favorable vs. Unfavorable)** | | | | | | | | | | | |
| **Univariate** | Not significant OS | Not significant 5y OS 42% vs.14% vs. 39% | Not significant | Significant on LRFS PM vs. all other sites 50% versus 83% | Not significant on OS, EFS (p=0.6809) | Not significant | Significant on OS, DSS | Significant on OS, DMFS | Significant on DSS, PFS | Significant (GU) OS, PFS | Not reported |
| **Mulivariate** | Not significant OS | Not significant | Not significant on DSS or DMFS | The only significant predictor factor on LRFS (PM site, p=0.003) | Not tested | Significant OS | Not significant | Significant on DMFS only | Significant on DSS, PFS | Not significant on PFS, OS | Not reported |
| **Histological subtype (E vs. non-E)** | | | | | | | | | | | |
| **Univariate** | Significant OS (p=0.0446) | Not significant 5y OS 43% vs. 29% vs. 26% | Not significant | *Significant 10y OS 27% vs. 44%, 10-year DMFS 32% vs. 58% | Significant 5y-OS 58.% vs. 30%; Significant EFS | Not significant | PRMS/ARMS significant OS, DSS | Significant DMFS, LRFS, OS | Not tested | Not reported | Not reported |
| **Mulivariate** | Significant only if Pleomorphic RMS was involved | Not significant OS | Not significant on DSS or DMFS | Not significant | Not tested | Significant OS (0.009), PFS | Significant OS, DSS only if Pleomorphic RMS was involved | Significant on DMFS and the only significant predictor of LRFS | Not tested | Not reported | Not reported |

| | | | | | | | | | | | |
|---|---|---|---|---|---|---|---|---|---|---|---|
| **Tumor invasiveness (T1 vs. T2)** | | | | | | | | | | | |
| **Univariate** | Significant OS | Significant 5y OS (p<0.0001) | Significant DSS (Local vs. distant) | Not reported | Significant 5y-OS 77% vs. 30.9% and EFS | Not significant | Significant (regional vs. Localized) on OS, DSS | Not reported | Not tested | Not reported | Not reported |
| **Mulivariate** | Significant OS (p<0.0001) | Not significant | Significant DSS | Not reported | Not tested | Significant OS (p=0.034), PFS | Significant on OS, DSS | Not reported | Not tested | Not reported | Not reported |
| **Tumor size (<5 vs. >5 cm)** | | | | | | | | | | | |
| **Univariate** | Not reported | Significant 5y OS 60% vs. 11% | 5y-DSS 52% vs. 22% | Signifiacnt OS, DFS, DMFS 10y OS 49% vs. 35% | Significant 5y-OS 75% vs. 30.4% and EFS | Not reported | Not reported | Not reported | Not tested | Not significant OS, RFS | Not reported |
| **Mulivariate** | Not reported | Not significant | Significant on MVA of DSS (p<0.03) BUT lost significance in MVA of LRFS or DMFS. | The only significant predictor on DFS, DMFS. | Not tested | Not reported | Not reported | Not reported | Not tested | Not significant OS, RFS | Not reported |
